# Maternal posture-physiology interactions in human pregnancy: a narrative review

**DOI:** 10.3389/fphys.2024.1370079

**Published:** 2024-07-19

**Authors:** Allan J. Kember, Jennifer L. Anderson, Natalyn E. Gorazd, Sarah C. House, Katherine E. Kerr, Paula A. Torres Loza, David G. Reuter, Sebastian R. Hobson, Craig J. Goergen

**Affiliations:** ^1^ Temerty Faculty of Medicine, Department of Obstetrics and Gynaecology, University of Toronto, Toronto, ON, Canada; ^2^ Shiphrah Biomedical Inc., Toronto, ON, Canada; ^3^ Larner College of Medicine, University of Vermont, Burlington, VT, United States; ^4^ Weldon School of Biomedical Engineering, Purdue University, West Lafayette, IN, United States; ^5^ Temerty Faculty of Medicine, Medical Education, University of Toronto, Toronto, ON, Canada; ^6^ Cardiac Innovations, Seattle Children’s, Seattle, WA, United States; ^7^ Temerty Faculty of Medicine, Institute of Medical Science, University of Toronto, Toronto, ON, Canada; ^8^ Maternal-Fetal Medicine Division, Mount Sinai Hospital, Toronto, ON, Canada

**Keywords:** gravity, posture, maternal, pregnancy, obstetrics, physiology, pathophysiology

## Abstract

There are several well-known medical conditions in which posture and gravity interact with natural history, including pregnancy. In this review, we provide a comprehensive overview of interactions between maternal posture and maternal physiology and pathophysiology at rest during pregnancy. We conducted a systematic literature search of the MEDLINE database and identified 644 studies from 1991 through 2021, inclusive, that met our inclusion criteria. We present a narrative review of the resulting literature and highlight discrepancies, research gaps, and potential clinical implications. We organize the results by organ system and, commencing with the neurological system, proceed in our synthesis generally in the craniocaudal direction, concluding with the skin. The circulatory system warranted our greatest and closest consideration–literature concerning the dynamic interplay between physiology (heart rate, stroke volume, cardiac output, blood pressure, and systemic vascular resistance), pathophysiology (e.g., hypertension in pregnancy), and postural changes provide an intricate and fascinating example of the importance of the subject of this review. Other organ systems discussed include respiratory, renal, genitourinary, gastrointestinal, abdominal, and endocrine. In addition to summarizing the existing literature on maternal posture-physiology interactions, we also point out gaps and opportunities for further research and clinical developments in this area. Overall, our review provides both insight into and relevance of maternal posture-physiology interactions vis à vis healthcare’s mission to improve health and wellness during pregnancy and beyond.

## Introduction

Life on Earth inherently brings with it a constant and unrelenting exposure that none of us can escape: the force of gravity. From the dawn of the anthropocene, gravity has exerted its effects on human behavior, health, and wellbeing. Beginning in the early 1800’s, numerous medical precedents have been described that highlight the interactions between gravity, body posture, physiology, and pathophysiology–congestive heart failure (Chen and Aronowitz, 2022), acute respiratory distress syndrome (Guérin et al., 2020), gastro-esophageal reflux disease (Dağlı and Kalkan, 2017), obstructive sleep apnea (Oksenberg et al., 2012), and sudden infant death syndrome (Jullien, 2021), to name a few. One hundred years later, researchers began to consider the impact of body posture and gravity on the physiology of pregnancy, particularly the mass effect of the gravid uterus on hemodynamics (Walker et al., 1933; Brigden et al., 1950; Pritchard et al., 1955; Holmes, 1960). After World War II, this interest continued into the 1960’s but largely dropped off until a publication by Stacey *et al.* in 2011 that demonstrated a significant association between sleeping supine in late pregnancy and stillbirth (Stacey et al., 2011). Riding a resurgence of curiosity and investigation around this topic in the last decade, we provide here a review of maternal posture-physiology interactions at rest from conception, throughout the trimesters, to the postpartum period in healthy pregnancies and those with comorbidities. In the current review, we deliberately exclude the impact of maternal posture on fetal physiology and pathophysiology because that was the subject of a separate review we recently published (Kember et al., 2024). Furthermore, we purposely use the term “posture” because its meaning has a closer association with the human body than “position”.

This review has wide relevance. As space travel becomes a tangible aspiration for humans in our generation and the next, the findings presented here have a burgeoning applicability beyond the confines of our planet’s gravitation to both supra-gravitational and sub-gravitational states. But even at home on Earth, advances in transportation and entertainment are subjecting common humans to supra-gravitational forces. While a commercial airliner pulls about 0.2-0.4G’s in the horizontal direction during take-off, electric vehicles, such as Tesla’s Model S, can pull upwards of 1.15G’s horizontally, which may have relevance in pregnancy. Equally intriguing is the speed-bump paradox – a long-used traffic safety measure in speed-calming zones, the speed-bump, due to the sudden acceleration it exerts on the vehicle’s occupants, has been recently investigated and posited as a potential public health risk in pregnancy (Irannejad Parizi et al., 2021). We hope this review spurs future research directions and has relevance to development of guidelines for posture in various activities encountered in pregnancy from preconception to postpartum. By this review, in addition to presenting work that has been completed, we also aim to highlight gaps in our knowledge with a view to future work. We hope this review is of interest to patients and their care providers–obstetricians, midwives, nurses, anesthetists, along with primary and allied care providers. As a team of clinicians, scientists, and engineers ourselves, we also hope this review will pique the interest of other professionals whose desire is to assist healthcare providers help their patients be well and experience healthy pregnancies.

## Methods

**FIGURE 1:**
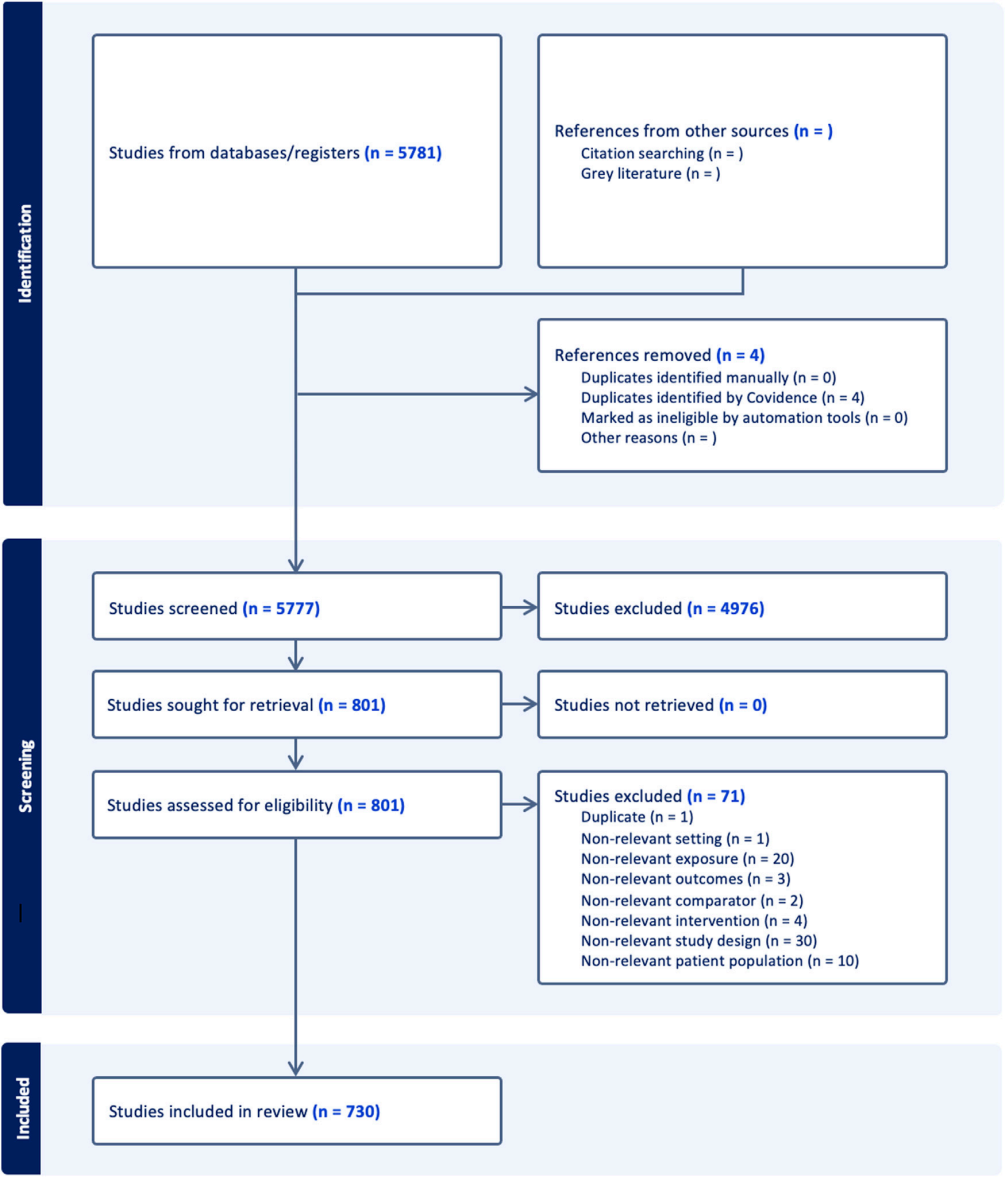
PRISMA flow diagram outlining the study selection process.

A comprehensive literature search was performed in August 2021 using MEDLINE. The keywords either described pregnancy or body posture (see Supplementary Material S1 – Search Strategy Keywords). The search was narrowed to results published between 1 January 1991 through 8 August 2021. Results were filtered to English language and human participants using MEDLINE’s built-in filters. The search yielded 5,781 publications. Duplicates (n = 4) were removed. Search results were then independently screened by four reviewers for adherence to inclusion and exclusion criteria. Included papers involved participants in the conception, antepartum, intrapartum, and/or postpartum periods and investigated the effect of maternal body posture on a maternal or fetal organ system or physiological/pathophysiological process. We excluded book chapters, editorials, commentaries, paper replies or responses, reviews (with the exception of systematic, meta-analysis, and Cochrane reviews), or if the publications were related to male fertility or infertility. This further refined the selection to a final number of 730 studies (Figure 1).

Note that for this review, we excluded the effect of maternal body posture on the fetus, umbilical cord, placenta, uterus, and cervix as this is the subject of a separate review we recently published (Kember et al., 2024). Perhaps due to its primary function being to resist the effects of gravity, the corpus of literature relating to the musculoskeletal system outnumbered, by far, the literature related to any other organ system and, as such, is discussed separately (see Supplementary Material S2 – Musculoskeletal Maternal Posture-Physiology Interactions). Of the 730 studies, 644 included maternal outcomes and are the subject of this review.

## Results

We observed that research articles of maternal posture-physiology interactions generally focused on 1) the effect of posture at rest on an organ system or 2) the interaction between posture and physiology during a given activity (e.g., exercise, labor and birth). For the purposes of maintaining a feasible scope, we excluded the latter category from this review and, instead, focused on maternal posture-physiology interactions at rest. We present the results from the perspective of different organ systems in a sequential manner.

### Neurologic

As pregnancy progresses, sympathovagal balance is shifted progressively from a higher vagal modulation in early pregnancy to a higher sympathetic modulation in late pregnancy (Chen et al., 1999a; Matsuo et al., 2007). This balance then returns to normal by 3 months’ postpartum, in which relief of aortocaval compression following delivery of the fetus may provide some contribution (Chen et al., 1999a). In late pregnancy, the autonomic response to changing position from upright to lying down supine is vagal attenuation with sympathetic predominance, and because the reverse effect is seen in non-pregnant persons, it is thought that aortocaval compression plays a role (Chen et al., 1999a). Further, in late pregnancy, the left lateral decubitus position, compared to the supine and right lateral decubitus positions, results in the least suppressed vagal activity and the least enhanced sympathetic activity (Kuo et al., 1997), and most closely approximates the balance of sympathovagal activity 3 months postpartum (Chen et al., 1999b). It is difficult to separate autonomic activity from resultant changes in hemodynamic parameters; therefore, we reserve the remainder of this discussion for the Autonomic subsection of the Cardiovascular section.

Postural orthostatic tachycardia syndrome (POTS) is characterized by an excessive increase in the heart rate upon standing along with orthostatic intolerance (Fedorowski, 2019). This rare and poorly-understood syndrome is related to autonomic nervous system dysfunction and primarily affects young women of child-bearing age. Patients with POTS experience a range of symptoms as a result of their inability to maintain peripheral vascular resistance in the upright position – a compensatory tachycardia ensues when they sit or stand upright to maintain blood pressure and peripheral perfusion, however syncopal episodes may remain frequent. Successful pregnancy, however, is possible in severe cases of POTS. In one case report including two patients, both experienced hyperemesis gravidarum and marked improvement in their POTS symptoms until about 6 months’ gestation, likely due to gestational volume expansion, after which episodes of syncope and tachycardia resulted in clinical deterioration requiring partial bed rest and wheelchair mobility (Glatter et al., 2005). One patient continued their midodrine five times daily throughout pregnancy. Both patients delivered healthy infants at 37 weeks by elective cesarean section. Intrapartum anesthetic management of POTS can be challenging due to hemodynamic instability (McEvoy et al., 2007). In postpartum follow-up, both patients experienced an improvement in their POTS symptoms compared to pre-pregnancy, which the authors hypothesized was due to physical conditioning during normal postpartum activities.

Neurocardiogenic syncope describes a group of clinical syndromes characterized by syncope as a result of inappropriate and often excessive autonomic reflex activity (Sutton and Petersen, 1995). There is one case in the literature of recurrent neurocardiogenic syncope with documented severe collapse of the IVC and profound sinus arrest after changing to the sitting position from the supine position in a patient at 34 weeks’ gestation (Huang et al., 2003). Another related syndrome, micturition syncope (temporary loss of consciousness while urinating), is extremely rare, predominantly affecting older males, likely due to the combination of increased vagal input from the bladder while voiding and the tendency to stand while voiding (Schiavone et al., 1991). Micturition syncope, however, has been described in the second trimester of pregnancy and is characterized by experienced multiple syncopal episodes related to urinary urgency, voiding in the sitting position, or a distended urinary bladder with or without travel motion (Sherer et al., 2005). It is felt that manifestation of this syndrome in the second trimester may be a result of the expanding uterus affecting bladder distension or sensory input from the lower urinary tract. Micturition syncope in pregnancy responds well to timed voiding (every 1–2 h) to avoid an overdistended bladder, especially prior to traveling (Sherer et al., 2005).

In the postpartum period, severe postural headache that is worse with sitting and improves with recumbent positioning is a common sign of a rare complication of epidural or spinal anesthesia known as a postdural puncture headache (PDPH). A PDPH has even been reported following spontaneous vaginal delivery in the absence of epidural or spinal anesthesia (Albayram et al., 2008). Mimics of PDPH have been reported, albeit rarely, including a postpartum cerebral venous thrombosis (superior sagittal sinus thrombosis) that was worse with the upright position and relieved by assuming the recumbent position (Chisholm and Campbell, 2001). Conversely, a headache in the postpartum period that is precipitated by Valsalva or recumbent posture and improves with upright posture, often described as a “thunderclap headache”, may indicate reversible cerebral vasoconstriction syndrome–a diagnosis that, if missed, could result in serious neurologic injury (Lenger and Barrier, 2014; Qubty et al., 2020).

### Ophthalmic

Body posture affects the eyes in both the pregnant and non-pregnant population. Intraocular pressure (IOP) is known to decrease in pregnancy (Qureshi et al., 1996). Within non-glaucomatous pregnancies in the third trimester, Kara *et al.* found that IOP is significantly lower in the sitting posture than in the supine, right lateral decubitus, and left lateral decubitus postures. The authors suggested this may be a result of increased episcleral venous pressure during pregnancy in the supine and lateral decubitus postures–noting that the IOP was not significantly different between the supine, right lateral decubitus, and left lateral decubitus postures (Kara et al., 2013). Ocular perfusion pressure (OPP), which is a function of the SBP, DBP, and IOP, is significantly different between these four postures with the highest OPP in the sitting posture followed by supine, right lateral decubitus, and left lateral decubitus. Because SBP, DBP, and mean BP were lowest in the left lateral decubitus posture, it follows that OPP was lowest in this posture. The measurements were taken at the 15th minute following adopting the posture and between 10a.m. and noon to minimize the effect of known diurnal IOP variation. Given the predilection for resting in the left lateral decubitus posture in pregnancy, these findings may have clinical implications such as an increased risk of ocular damage being sustained in postures with lower OPP. In the intrapartum and postpartum periods, posture (seated, supine, and left lateral) does not affect IOP in normotensive patients nor in those with preeclampsia, but those with preeclampsia have a higher IOP than their normotensive counterparts (Giannina et al., 1997).

### Circulatory system

Significant changes occur in the circulatory system from conception through postpartum to accommodate the growing fetus. These changes include, but are not limited to, blood volume expansion, increased heart rate, increased cardiac output, increased blood pressure, and reduced systemic vascular resistance. Furthermore, these changes are mediated in part by alterations in responsiveness to pressors (e.g., angiotensin, norepinephrine), changes in endothelial prostacyclin and nitric oxide production, and possible contributions of pregnancy hormones (e.g., estrogen, progesterone) to hemodynamics. Here, we discuss the impact of posture on the circulatory system, with a focus on three systems: 1) cerebrovascular, 2) cardiac, and 3) peripheral vascular.

#### Cerebrovascular

Cerebral blood flow increases and cerebral vascular resistance decreases in pregnancy. Cerebral blood flow velocity is known to be higher in pregnancies with preeclampsia and chronic hypertension compared to those who are normotensive (Williams and MacLean, 1994a; Williams and MacLean, 1994b; Zatik et al., 2001). However, cerebral vessels in pregnancies with preeclampsia are thought to be vasospastic and respond differently to changes in posture as compared to those who are normotensive or chronically hypertensive (Williams and MacLean, 1994a; Williams and MacLean, 1994b; Zatik et al., 2001). Cerebral blood flow response to a change in posture (from left lateral to sitting) is also different in severe *versus* mild preeclampsia and in preeclampsia *versus* gestational hypertension (GH) (Chipchase et al., 2003). Severe cases of preeclampsia were more likely to see an increase in cerebral blood flow with a change in posture, while mild preeclampsia and GH cases typically saw a decrease. Williams and MacLean also found cerebral blood flow velocity rises significantly in pregnancies with preeclampsia when changing from left lateral decubitus to sitting and additionally noted no significant change in mean blood pressure (Williams and MacLean, 1994b). In contrast, pregnancies with chronic hypertension saw no significant differences in cerebral blood flow in any of the postures. Zatik *et al.* found an increase in mean arterial blood pressure and cerebrovascular resistance after changing from left lateral to supine, along with a decrease in mean blood flow velocity in the middle cerebral artery of pregnancies with preeclampsia (Zatik et al., 2001). The cerebrovascular resistance increased in both normotensive and preeclamptic pregnancies; however, the increase was larger in those with preeclampsia, while the cerebral blood flow index remained constant. A greater autoregulatory response was required to maintain cerebral blood flow when cerebral perfusion pressure increased. These results suggest pregnancies with preeclampsia have alterations in autoregulation of cerebral circulation, but further research is needed to elucidate the pathophysiology. While most tests fail to see clinical utility, the evidence is clear that postural changes in pregnancy present a hemodynamic challenge for the cerebrovascular system, and especially so in the setting of preeclampsia where hemodynamic dysfunction is more apparent with postural changes.

Supine hypotensive syndrome (SHS), discussed further below, is a widely recognized disorder of maternal circulation resulting from hemodynamic changes imposed by compression of the inferior vena cava (IVC) by the gravid uterus when supine. Ikeda *et al.* demonstrated that cerebral blood flow volume in pregnancies with SHS and subclinical SHS when supine is significantly lower than in pregnant controls (Ikeda et al., 1992). Pregnancies with SHS also had a decrease in mean blood flow velocity in the internal carotid artery compared to pregnant controls without SHS in the supine posture. When mean velocity decreased below 10 cm/s, participants experienced dizziness, nausea, and syncope reflecting cerebral hypoperfusion. Symptoms of palpitations and shortness of breath were experienced at mean blood flow velocities above 10 cm/s, suggesting an association with tachycardia rather than hypoperfusion. Cerebral hypoperfusion was not found in any participant while in the sitting posture, reinforcing the safety of this position for all pregnancies.

#### Cardiac

We approach the cardiac system by considering the interplay between body posture and hemodynamics first and, subsequently, the interplay between body posture and autonomic control of cardiac function and peripheral vascular tone.

##### Hemodynamic

Cardiac output (CO) is a function of the heart rate (HR) and stroke volume (SV) – we consider these three parameters first, followed by an exploration of blood pressure (BP) and systemic vascular resistance (SVR) and provide a summary table of the impact of maternal posture on hemodynamics in Table 1. Finally, we provide a detailed discussion of a clinical entity that brings all of these concepts together: supine hypotensive syndrome (SHS).

**TABLE 1:** Summary table of impact of maternal posture on hemodynamics during pregnancy for five main maternal postures.

	Supine	Left lateral	Right lateral	Sitting	Upright
Heart rate	↑^a^	Ref	↑^b^	↑^c^	↑^d^ ^,^ ^e^ ^,^ ^f^ ↑↑^g^
Stroke volume	Ref			↑^h^	↓^i^
*In third trimester*	↓↓^j^	Ref	↓	↓	
Cardiac output		Ref		↓^k^	↑↑^l^ ↑^m^ ↓^k^
	Ref	↑^n^		↑^d^ ^,^ ^h^ ^,^ ^k^	↑^l^ ↑↑^m^ ↑↑↑^k^ ↓ ^d^ ^ , ^ ^o^
Blood pressure					
*SBP*	↑^p^	Ref		↑^p^	↑^q^
	Ref				↓^r^
	Ref^s^	↓	↓		
*DBP*		Ref			↑^t^
	Ref				↑^u^
*MAP*	Ref	↓^p^		↑^v^ ↓^w^	
Systemic vascular resistance	Ref				↑^d^ ^,^ ^x^
	↑^y^	↓		Ref	↑

Contradictory findings are indicated by red text.

Abbreviations: Ref indicates reference posture. SBP, indicates systolic blood pressure; DBP, indicates diastolic blood pressure; MAP, indicates mean arterial pressure.

^a^ In pregnancies with supine hypotensive syndrome.

^b^ Four beats-per-minute faster.

^c^ In pregnancies affected by obesity.

^d^ In normotensive pregnancies.

^e^ This is the baroreflex response, and it is attenuated as gestation advances.

^f^ In pregnancies with gestational hypertension.

^g^ In pregnancies with severe preeclampsia.

^h^ Attenuated in pregnancies that developed preeclampsia or GH.

^i^ Attenuated as gestation advances, except in pregnancies with preeclampsia, with one study showing a non-significant increase in SV from supine to standing in the third trimester.

^j^ Significant reduction in SV when supine compared to left lateral was not observed in pregnancies affected by overweight and obesity.

^k^ Third trimester

^l^ First trimester

^m^ Second trimester

^n^ Occurs as early as 20 weeks’ gestation, but not in pregnancies affected by obesity.

^o^ Attenuated as gestation advances, except in pregnancies with preeclampsia.

^p^ At five minutes after posture change.

^q^ At one minute after posture change; greatest increase in normotensive pregnancies, followed by pregnancies with GH, then pregnancies with preeclampsia.

^r^ At three minutes after posture change; attenuated as gestation advances in normotensive pregnancies but not in pregnancies with hypertension (chronic or preeclampsia).

^s^ Supine posture with 15° of left lateral tilt.

^t^ At one minute after posture change; increase in pregnancies with GH significantly greater than in pregnancies with preeclampsia.

^u^ At three minutes after posture change; in normotensive pregnancies and attenuated as gestation advances.

^v^ At three minutes after posture change; in normotensive pregnancies at 35-37 weeks’ gestation, attenuated with increasing BMI.

^w^ At three minutes after posture change; in pregnancies at 35-37 weeks’ gestation that subsequently developed preeclampsia.

^x^ Attenuated as gestation advances, except in pregnancies with hypertension (not attenuated).

^y^ At five minutes after posture change; in normotensive pregnancies, and attenuated in pregnancies with preeclampsia or GH.

###### Heart rate

In normotensive pregnancies in the second and third trimesters, changing posture from a horizontal posture to standing increases maternal HR between 14% and 30% (Clark et al., 1991; Hohmann and Künzel, 1991; Schneider et al., 1993). This phenomenon, known as the baroreflex response, is diminished as pregnancy progresses (Hohmann and Künzel, 1991), especially after 20–24 weeks (DSilva et al., 2014; Miyake et al., 1993). In pregnancies with preeclampsia, however, this HR increase is significantly greater than normotensive pregnancies, pregnancies with GH, and non-pregnant controls, indicating autonomic dysfunction (Dyer et al., 2004). We point out a unique study by Schneider *et al.* that noted a more marked increase in a subset of participants who had cyclic HR accelerations while standing. It is thought that these cyclic HR accelerations are a result of cyclic preload changes secondary to autotransfusion from uterine contractions that occur predominantly when upright as a result of compression of the pelvic vessels by the gravid uterus–this phenomena has been dubbed “orthostatic uterovascular syndrome”. Cyclic accelerations in the maternal HR in phase with uterine contractions when upright have been documented to occur as early as 24 weeks’ gestation (5% prevalence) with a peak prevalence of 71% reached at 38 weeks (Schneider et al., 1993).

Maternal HR is higher in the right lateral and left lateral postures compared to sitting in term, non-labouring pregnancies (Armstrong et al., 2011). Carson *et al.* found, however, the prevalence of sinus tachycardia (HR ≥ 100 bpm) to be higher in the sitting posture (39%) compared to the left lateral recumbent posture (29%) between 32–40 weeks. This effect was particularly seen in pregnancies affected by obesity where prevalence reached 58% in the sitting position, compared to 29% in pregnancies without obesity (Carson et al., 2002). We hypothesize that due to the higher intra-abdominal pressures experienced due to the pannus in obesity, pregnancies affected by obesity may be more sensitive to postural changes that result in an orthostatic challenge. On the left side, they found no difference in sinus tachycardia prevalence in pregnancies with and without obesity (32% *versus* 27%, respectively).

When changes between horizontal postures are considered, large changes seem to affect HR whereas small variations in postures (e.g., tilting) do not. Armstrong *et al.* demonstrated that maternal HR is higher in the right lateral and left lateral postures compared to supine with 15° of left tilt in term non-labouring pregnancies (Armstrong et al., 2011). We add that a slower HR when supine, at least initially, is intuitive because, due to reduced venous return and cardiac preload when supine, a slower HR allows for increased cardiac chamber filling time and maintenance of stroke volume. This effect is dynamic and transitory as increased preload (e.g., passive leg raise while lying on the left side) normally results in a slight reduction in HR in the third trimester (Guy et al., 2018). However, in supine hypotensive syndrome (discussed in detail below), HR increases significantly when supine compared to left lateral in the third trimester (Humphries et al., 2020). When left is compared to right lateral in term, non-laboring pregnancies, the average HR is four beats-per-minute slower in the left lateral position, and periodic HR changes due to uterine activity are significantly more common (85% vs 11%) when in the right lateral posture compared to the left (Ibrahim et al., 2015). Dennis *et al.*, in a rare study that included prone position in term pregnancies, were unable to find a clinically significant difference in HR after 5 minutes of rest in the prone posture compared to the left lateral posture, neither in normotensive nor preeclamptic pregnancies (Dennis et al., 2018a). Regarding small variations in horizontal postures, Ellington *et al.* did not find a significant difference in maternal HR (measured 3 minutes after assuming each posture) in the supine posture with various angles (0°, 5°, 10°) and directions (left, right) of lateral tilt. This study, however, included pregnancies from 25 through 40 weeks, which may have diluted their results as aortocaval compression from the gravid uterus at 25 weeks is significantly different than that occurring at 40 weeks (Ellington et al., 1991).

###### Stroke Volume

When changing position from a horizontal posture (left lateral or supine) to standing, there is a significant reduction in stroke volume (SV) (Schneider et al., 1993). This reduction in SV with standing is attenuated as pregnancy advances, especially beyond 20–24 weeks (Miyake et al., 1993; Del Bene et al., 2001). Pregnancies with preeclampsia, however, continue to experience a profound decrease in SV upon standing beyond 32 weeks in comparison to pregnancies with chronic hypertension, reflecting autonomic dysfunction (Miyake et al., 1993).

Atrial natriuretic peptide (ANP) is a peptide hormone secreted by the atria of the heart in response to atrial distension. ANP is a diuretic, natriuretic, and vasorelaxant and antagonizes the renin-angiotensin system at multiple levels. Sala *et al.* investigated postural effects on ANP and SV across the trimesters and in the postpartum period and found an attenuation of supine plasma ANP across the first, second, and third trimesters with the lowest value occurring in the postpartum period (Sala et al., 1995). The highest supine plasma ANP occurring in the first trimester was presumably due to physiologic volume expansion of pregnancy, and this was corroborated by the highest supine SV also occurring in the first trimester. The upright plasma ANP values were significantly lower than the supine plasma ANP values during every trimester with the difference between standing ANP *versus* supine ANP being attenuated as gestation advanced, which paralleled the changes in standing SV *versus* supine SV across the trimesters. In the first trimester and postpartum period, there was a significant decrease in SV when changing position from supine to standing. In the second trimester, this decrease was non-significant. In the third trimester, there was a non-significant increase in SV when changing position from supine to standing. These parallel attenuations in “standing ANP *versus* supine ANP” and “standing SV *versus* supine SV” as gestation advances may be due to initial physiologic hypervolemia of pregnancy followed by, as gestation advances, increasingly impaired venous return to the right side of the heart via compression of the IVC by the increasingly gravid uterus when supine and relief of this compression upon standing (Sala et al., 1995).

Changing from a horizontal posture (left lateral or right lateral) to sitting has been found to result in a reduced SV in term pregnancies (Armstrong et al., 2011). On the contrary, changing from the supine position to sitting has been found to increase SV at 35–37 weeks, presumably indicating preload responsiveness from relief of IVC compression, but this effect was curiously attenuated in pregnancies that subsequently developed preeclampsia or GH (Guy et al., 2018).

In the third trimester, considering changes between horizontal positions, it is well documented that SV is highest in the left lateral position (Bamber and Dresner, 2003; Tamás et al., 2007), followed by the right lateral position, supine position (Carpenter et al., 2015), supine position with 15° of left tilt (Armstrong et al., 2011)., with the lowest SV being in the supine position with right tilt (5°–12.5°) (Bamber and Dresner, 2003). In a serial cardiac MRI study of normotensive, nulliparous (n = 14 normal weight, n = 9 overweight/obese) patients by Nelson *et al.*, posture did not alter SV during early pregnancy (Nelson et al., 2015). However, when they imaged these patients at 26–30 weeks and 32–36 weeks, they observed that the significant reduction in SV when supine compared to left lateral in normal weight subjects did not occur in pregnancies affected by overweight or obesity (Nelson et al., 2015), possibly a protective effect (vis-à-vis maintenance of preload) inherent in the latter group either via intra-abdominal adipose tissue preventing aortocaval compression or a more efficiently developed collateral venous bypass. Positive effects on venous return (preload) and SV of the left lateral posture compared to the supine posture are apparent on cardiovascular MRI as early as 20 weeks’ gestation and persist throughout late pregnancy (Rossi et al., 2011).

Overall, SV is a function of venous return to the right side of the heart (i.e., cardiac preload). In pregnancy, challenges to maintenance of SV are those that challenge cardiac preload, including orthostatic stress (e.g., transition to standing), IVC compression (e.g., from supine posture), and intravascular volume contraction (e.g., preeclampsia). During the first half of pregnancy, physiologic volume expansion helps to maintain preload and SV in the face of orthostatic stress. With advancing gestation, however, the supine posture, and concomitant IVC compression by the gravid uterus, continues to challenge maintenance of preload and SV.

###### Cardiac output

We have previously noted changes in HR and SV with changes in posture and now turn our attention to the product of these two variables: cardiac output (CO).

Upon changing from a horizontal posture to standing, the effect on CO is trimester dependent. In the first trimester, there is an increase in CO upon standing (DSilva et al., 2014; Del Bene et al., 2001), and this increase in CO is attenuated in the second trimester (Del Bene et al., 2001), whereas others have reported no changes (Sala et al., 1995) or a decrease (Schneider et al., 1993). In the third trimester, there is a decrease (11%–18%) in CO upon changing from the left lateral, right lateral, or sitting position to standing (Clark et al., 1991; Sorensen et al., 1992; Miyake et al., 1993; Schneider et al., 1993), and only one group reported no change in CO when changing from left lateral to standing (Del Bene et al., 2001). Of note, the horizontal starting posture seems to be important: when considering a change from supine to standing in the third trimester, Sala *et al.* (Sala et al., 1995; DSilva et al., 2014) both reported an increase in CO, not a decrease as observed when starting out from a lateral posture. Also notably, D’Silva *et al.* reported the increment in CO when changing from supine to standing increases from the first through the third trimester (DSilva et al., 2014). This is intuitive because upon relief of the IVC compression occurring when supine, the venous congestion upstream of the compression would be released and seen as a bolus of preload to the right side of the heart, increasing CO. However, these results were contradicted by Miyake *et al.* who, in considering a potential interaction with blood pressure, found that CO decreases when changing from supine to standing in normotensive pregnancies. They found this effect to be attenuated as pregnancy advances, especially after 20–24 weeks, but not in preeclampsia, where compared to pregnancies with chronic hypertension the decrease in CO from supine to standing continued to be profound after 32 weeks (Miyake et al., 1993). We offer that this latter finding may be a result of increased sensitivity to orthostatic stress due to widespread vasoconstriction and volume contraction seen in preeclampsia.

In the third trimester, changing from horizontal postures to sitting reflect similar physiology as the horizontal to standing transition: the CO decreases when changing from the right or left lateral postures to sitting (Armstrong et al., 2011), and the CO increases when changing from the supine posture to sitting in normotensive pregnancies, but this is attenuated in pregnancies that subsequently develop preeclampsia or GH (Guy et al., 2018).

In early pregnancy and by 12 weeks’ postpartum, CO is not different between the left lateral and supine postures (Nelson et al., 2015). As early as 20 weeks gestation, however, CO is significantly higher in the left lateral posture compared to the supine posture (Rossi et al., 2011), but this has not been demonstrated in pregnancies affected by obesity (Nelson et al., 2015). In the third trimester, CO is approximately 9%–16% higher in the left lateral compared to the supine posture, (Clark et al., 1991; Park and Hidaka, 1991; Rossi et al., 2011; Lee et al., 2012; Nelson et al., 2015; Humphries et al., 2019) but, again, this effect was not observed in pregnancies affected by obesity in a small study where the authors hypothesized that the absence of this effect may be due to a cushioning effect of excess intra-abdominal adipose tissue limiting aortocaval compression and subsequent hemodynamic changes (Nelson et al., 2015). When horizontal postures with lateral tilt are considered, at term, CO is 8.1% higher in the right lateral posture compared to supine with 15° of lateral tilt to the left (Armstrong et al., 2011). Further resolution is provided by Bamber and Dresner who used an operating table to achieve lateral tilt postures (0°, left 5°, left 12.5°, right 5°, and right 12.5°) (Bamber and Dresner, 2003). Compared with the left lateral posture, which had the highest CO, there was a mean 17% reduction in CO when supine with tilt to the right, which had the lowest CO. They found that increasing the amount of lateral table tilt from supine to the left or right did not have a significant effect on CO (Bamber and Dresner, 2003); however, this non-significant effect may be explained by Kundra *et al.* who showed that moving to the left lateral tilt posture from the supine posture is not as effective in relieving aortocaval compression as when moving to this same posture from the left lateral posture (Kundra et al., 2012). In summary, it appears that not all supine with left lateral tilt postures are created equal–it depends on the posture from which one starts out prior to arriving there.

##### Blood Pressure

###### Measurement

Maternal posture affects blood pressure (BP) measurement–the accuracy of which is critical for diagnosis and monitoring of important complications in pregnancy. In recent decades, considerable variation in maternal posture during clinical BP measurement was recognized and raised as a concern in the literature (Brown and Simpson, 1992). The importance of posture is reflected in the recommendation of several professional societies that the pregnant patient should be sitting upright and the sphygmomanometer cuff positioned at the level of the heart (Helewa et al., 1997; Magee et al., 2014; Gestational Hypertension and Preeclampsia: ACOG Practice Bulletin, 2020).

Some clinical scenarios call for measurement of BP while the patient is in a horizontal posture. There is controversy regarding whether hydrostatic effects are present and clinically significant, which we will not explore further here (Goldkrand and Jackson, 1997; Hallak et al., 1997; Kinsella, 2006). The American College of Obstetrics and Gynecology’s 2020 practice bulletin on GH and preeclampsia states that for hospitalized patients, BP can be measured in the left lateral recumbent position with the patient’s arm at the level of the heart (Gestational Hypertension and Preeclampsia: ACOG Practice Bulletin, 2020).

Overall, inclusion of maternal posture in recommendations pertaining to BP measurement in pregnancy draws from the field of fluid mechanics and lends credence to the importance of the topic of this review.

###### Changes with posture

**FIGURE 2:**
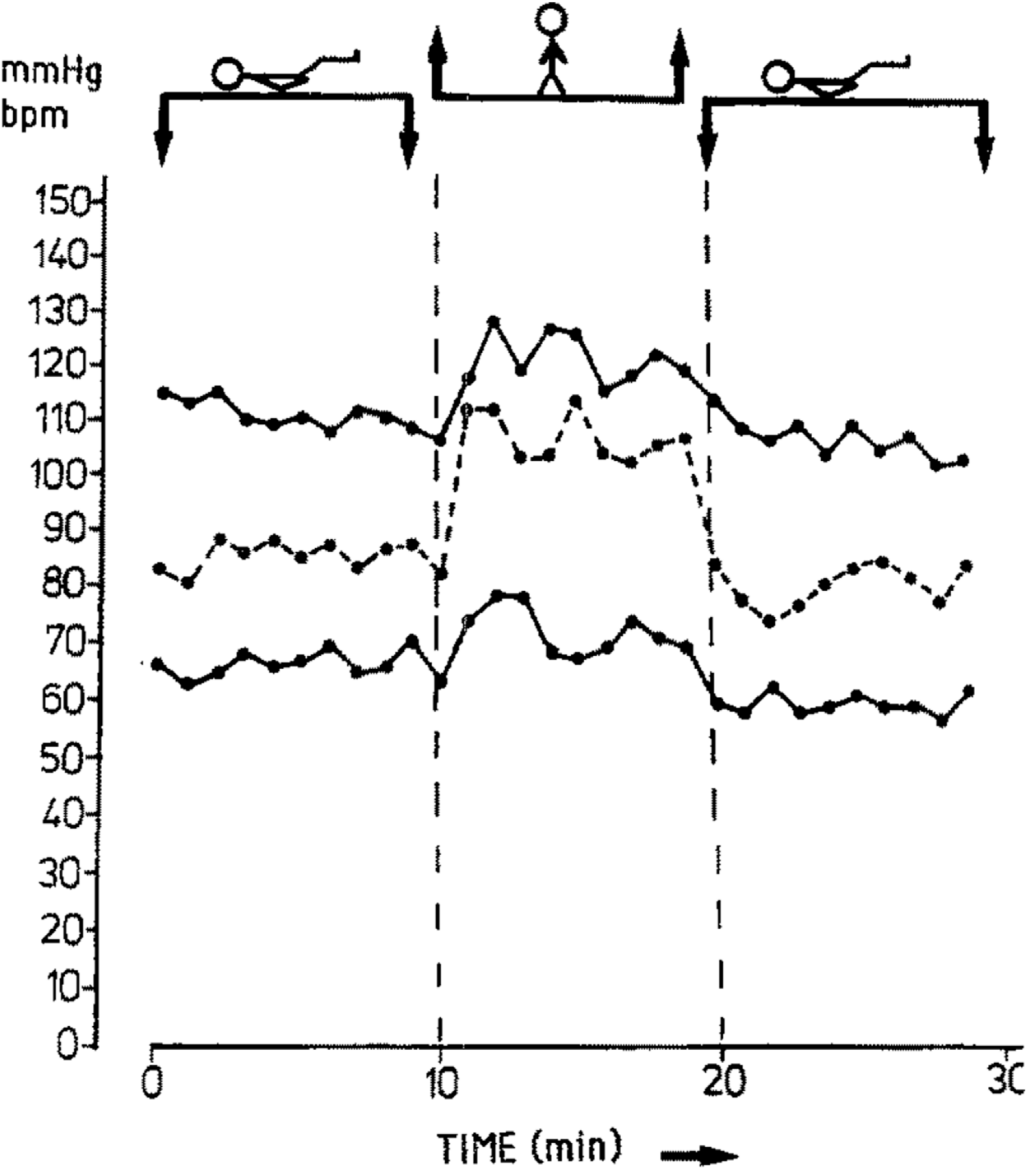
Changes in systolic blood pressure (upper line), diastolic blood pressure (lower line), and heart rate (dashed line) in one subject as an example of experimental design. Note that while the stick figure appears supine in the figure above, the participant actually started out in the left lateral recumbent posture, then changed to the standing posture, then returned to the left lateral decubitus posture. Used with permission from Springer Nature (Hohmann and Künzel, 1991)

When changing from a horizontal posture to standing, the change in BP depends on the specific horizontal posture one starts out in, the timing of BP measurement, and the presence of comorbid conditions. In pregnancies in the second and third trimester and non-pregnant controls, Hohmann and Künzel measured systolic BP (SBP) and diastolic BP (DBP) at 1 minute intervals for 10 minutes after changing from the left lateral posture to standing (Hohmann and Künzel, 1991). In short, they reported an approximate threefold greater BP response to standing in the second and third trimesters compared to non-pregnant controls. Of note, we direct readers to Figure 2, below, from Hohmann and Künzel’s paper, which presents a single participant’s measurements and highlights the dynamic nature of SBP and DBP from minute to minute while resting in the left lateral posture, upon standing, and then returning to the left lateral posture–we contend that this dynamicity may account for some of the discrepancies in the following discussion.

Dyer *et al.* provided insight into immediate BP changes by measuring BP at 1 minute following standing up from resting in the left lateral posture in normotensive pregnancies, pregnancies with GH, pregnancies with preeclampsia, and non-pregnant controls. At 1 minute after standing, there was a significantly greater mean increase in SBP in normotensive pregnancies compared to the other three groups. Pregnancies with preeclampsia had a significantly lesser mean increase in SBP compared to pregnancies with GH. However, as for DBP, there was a significantly greater mean increase in pregnancies with preeclampsia and in pregnancies with GH compared to the non-pregnant controls, but the mean increase in DBP in pregnancies with preeclampsia was significantly lesser than in pregnancies with GH (Dyer et al., 2004). Miyake *et al.* provided insight with regard to intermediate-term changes in BP by measuring BP 3 minutes following standing up from resting in the supine posture for 15 minutes. They found that SBP decreased, and this effect was attenuated as pregnancy advanced in normotensive pregnancies, especially after 20–24 weeks (Miyake et al., 1993). This finding was in contrast to their findings in pregnancies with hypertension (chronic or preeclampsia) where the decrease in SBP was not attenuated in late pregnancy (after 32 weeks). With regard to DBP, they found that it increases in normotensive pregnancies when changing from supine to standing, and this effect was attenuated as pregnancy advanced, especially after 20–24 weeks (Miyake et al., 1993). Van Katwijk and Wladimiroff presented opposite findings, describing a statistically significant decrease in SBP and DBP when moving from the standing to the supine position (van Katwijk and Wladimiroff, 1991); however, they did not provide any details as to the timing of their BP measurements in relation to assuming each position, which may explain this discrepancy. Note that the morphology of the SBP plot in Figure 2, while only representing one participant’s data, can be appreciated to roughly approximate Dyer *et al.*‘s finding at 1 minute (increase in SBP) and Miyake *et al.*‘s findings at 3 minutes (decrease in SBP), which likely represents an overshoot-and-undershoot pattern as the SBP reaches its new steady state.

Three studies have considered the changes in BP when changing from a horizontal posture to sitting. One study of term, non-labouring pregnancies showed that SBP (measured at 5 minutes after assuming the posture) is higher in the supine posture and when sitting with the hips and neck flexed compared to the right lateral and left lateral postures (Armstrong et al., 2011). Another study found a slight increase in the mean arterial pressure (MAP; measured for 120 s at 3 minutes following the position change) after changing to sitting from the supine posture in normotensive pregnancies at 35–37 weeks. This effect was attenuated with increasing BMI, and the opposite effect was seen in pregnancies that subsequently developed preeclampsia, potentially reflecting impaired preload reserve and/or dysregulated vascular tone (Guy et al., 2018). A third study in non-labouring, non-anaesthetised, healthy, term pregnancies found no significant difference in SBP or DBP between the left lateral decubitus posture, ramped posture with a wedge (for lateral pelvic tilt), and ramped posture alone (Dennis et al., 2018b).

When changing between horizontal postures, orthostatic forces are eclipsed by the compressive force of the gravid uterus on the inferior vena cava and the renal veins (when supine or near supine), which likely has the greatest effect on hemodynamics and hormonal regulators of BP in these postures. Armstrong *et al.* found that SBP (measured at 5 minutes following posture change) is lower in the flexed right lateral and flexed left lateral postures compared to the supine posture with 15° of left lateral tilt in healthy, term, non-laboring pregnancies (Armstrong et al., 2011); however, they measured BP via a noninvasive arterial BP cuff in the non-dependent arm, and it is unclear if they corrected for hydrostatic effects since indirect measurement of BP from the non-dependent arm in the lateral position is known to underestimate the true BP (Kinsella, 2006). That said, however, these data corroborate Goldkrand and Jackson who reported a lower MAP (measured at 5 minutes following posture change) in the lateral posture compared to the supine posture in pregnancies with normotension as well as those with chronic hypertension and preeclampsia (Goldkrand and Jackson, 1997). In term, non-labouring pregnancies via the use of invasive (radial artery line) systolic arterial pressure (SAP) measured for 3 minutes in the supine and left lateral postures, Erango *et al.* identified two distinct clusters of patients with clinically distinct differences in SAP response after changing from left lateral to the supine posture. One cluster was characterized by an initial sharp drop in SAP (10 mmHg drop in the first 100 s followed by leveling for the remaining 100 s), whereas the other cluster showed an initial increase in SAP (5 mmHg increase in the first 50 s followed by a slow decrease for the remaining 150 s). The former cluster, after spinal anesthesia, showed a drop in BP that was more rapid and greater than for the latter cluster. The former cluster also had a slightly higher mean CO after spinal anesthesia (Erango et al., 2018). The authors contended that the phenomenon observed in the former cluster is predominantly a result of aorto-caval compression when supine but did not provide further hypotheses regarding the underlying physiology or anatomy. Since aortocaval compression in the supine posture is universal in term pregnancies, we submit that, “all aorto-caval compression is not created equal,” that is, variability in collateral venous anatomy begets variability in physiology, which we will discuss more in the following sections.

Ellington *et al.* investigated BP changes in the supine posture with varying degrees of left and right lateral tilt achieved by an operating room table in non-laboring pregnancies between 25 and 40 weeks. Measured 3 minutes following the change in posture, they did not find any significant differences in BP between the supine, 5° right lateral tilt, 10° right lateral tilt, 5° left lateral tilt, 10° left lateral tilt, and 10° left lateral tilt with a 10 cm wide wedge (rolled blanket) placed under the right hip (Ellington et al., 1991).

Oliveira *et al.*, studying healthy pregnancies at 20–37 weeks, reported a significant decrease in SBP at 6 minutes after changing from left lateral to the prone posture (Oliveira et al., 2017). In a larger study, Dennis et al., did not observe a clinically significant change in SBP (measured at 5 minutes) in their group of healthy, normotensive pregnancies at term when changing from left lateral to the prone posture; however, they reported a significant reduction in SBP in pregnancies with preeclampsia, with one-third of these pregnancies experiencing a ≥10 mmHg reduction (Dennis et al., 2018a). The potential clinical benefit of this finding is obvious. Pregnancy cushions or activities such as swimming that allow transient prone positioning may reduce the degree of left renal vein outflow obstruction and, thereby, optimize renal hemodynamics and resultant hormonal modulation of BP. We discuss this further in the Renal section, below.

###### Systemic vascular resistance

Compared to pre-pregnancy, the systemic vascular resistance (SVR) in the left lateral posture decreases in the second trimester and decreases further in the third trimester (Del Bene et al., 2001). The SVR increases when changing from a horizontal (left lateral, supine) posture to standing in normotensive pregnancies (Clark et al., 1991; Sorensen et al., 1992; Miyake et al., 1993; Del Bene et al., 2001), but this increase is attenuated as pregnancy advances (Del Bene et al., 2001), especially after 20–24 weeks (Miyake et al., 1993). However, Miyake et al. found this to be in contrast to pregnancies with hypertension, where this SVR increase is not attenuated in late pregnancy. Furthermore, they reported the increase in SVR from supine to standing to be profound in pregnancies with preeclampsia after 32 weeks, compared to pregnancies with chronic hypertension (Miyake et al., 1993).

Sorensen *et al.*, studying pregnancies with normotension and pregnancies with hypertension in the third trimester, reported an increase in SVR in all participants when changing from sitting to standing (measured 5 minutes following the change in posture) (Sorensen et al., 1992). The change in SVR when changing to the sitting posture from a horizontal posture depends on initial posture. Sorensen et al. also reported an increase in SVR in all participants when changing from left lateral recumbent posture to sitting (Sorensen et al., 1992). Guy *et al.* reported a decrease in the SVR at 5 minutes after changing to sitting from supine in normotensive pregnancies in the third trimester–an effect that was attenuated in pregnancies with preeclampsia and gestational hypertension (Guy et al., 2018). Similarly, a reduction in SVR was seen after a passive leg raise was performed in the left lateral position (Guy et al., 2018). Taken together, the findings of Sorensen *et al.* and Guy *et al.* indicate that preload is improved or restored in the sitting posture compared to supine.

##### Clinical correlate: supine hypotensive syndrome

If the higher-pressure arterial system is impacted by changes in maternal posture, surely the lower-pressure venous system is more subject to the changes in posture and the force of mass and gravity. Indeed, supine hypotensive syndrome (SHS) is a “case in point” of how gravity and posture affect anatomy and, subsequently, physiology. SHS is defined as a drop in systolic blood pressure of 15–30 mmHg or an increase in heart rate of 20 bpm, with or without symptoms, after assuming the supine position (Humphries et al., 2020). In the third trimester, SHS has been reported with a prevalence of 4.5%-10% (Ellington et al., 1991; Schneider et al., 1993; Moffatt and van den Hof, 1997; Tamás et al., 2007). In SHS, the gravid uterus compresses the inferior vena cava (IVC), which, in some people, results in a decrease in venous return to the heart, a decrease in cardiac preload, and subsequently a decrease in stroke volume. The reduction in stroke volume often results in a compensatory increase in heart rate (Humphries et al., 2020) but ultimately results in a decrease in cardiac output and may lead to inadequate perfusion of vital organs such as the brain, resulting in symptoms of dizziness, nausea, neurocardiogenic syncope, and in extreme cases, death (De-Giorgio et al., 2012). Here, we consider anatomical aspects of SHS followed by physiological aspects.

In the supine posture in the third trimester, IVC compression is universal but SHS is not. Only one study challenges the assertion that IVC compression is universal in the supine position (Fields et al., 2013); however, they examined patients recruited from a labor ward (but not in active labor) and did not report on uterine activity, which, if present, would relieve IVC compression in the supine position (discussed later). A sonographic study by Ryo *et al.* in which the IVC was measured in non-pregnant individuals and pregnant individuals throughout gestation (4–40 weeks) demonstrated an initial increase in IVC size in the supine posture from the non-pregnant state to early pregnancy, likely due to the physiologic increase in blood volume that occurs in early pregnancy (Ryo et al., 2004). Despite this initial increase, as pregnancy advanced, IVC size was negatively related to gestational age in the supine posture, but positively related in the left lateral posture, emphasizing that with increasing gestational age (and increasing uterine volume), IVC compression becomes a greater concern. Indeed, several studies report an increase in IVC dimensions during late-pregnancy in the left lateral posture compared to supine (Kienzl et al., 2014; Higuchi et al., 2015; Saravanakumar et al., 2016; Fujita et al., 2019).

In two similar magnetic resonance imaging studies, Higuchi *et al.* and Fujita *et al.* describe a significant increase in IVC volume with a 30° left lateral tilt compared to the supine posture (Higuchi et al., 2015; Fujita et al., 2019). In both studies, a smaller left lateral tilt angle of 15° did not differ significantly from the supine posture. Fujita’s group additionally compared IVC volume in a right lateral 15° and 30° tilt, finding no significant differences between either posture and the supine and 15° left lateral tilt posture (Fujita et al., 2019). In short, change in IVC volume was only significant with 30° of left lateral tilt, suggesting that laterality and degree of tilt matters. Another study supports this and provides further insight: in sum, they found significantly different hemodynamic states in the femoral vein and artery when moving to the same posture (15° left lateral tilt) from two different initial postures (left lateral and supine). Their findings may explain conflicting results regarding the 15° left lateral tilt posture seen in other studies (Kundra et al., 2012). The take-away is that not all paths to 15° of left lateral tilt produce equal hemodynamic states–it depends on your starting posture. Furthermore, referring back to the findings of Higuchi *et al.* and Fujita *et al.*, another key point is that *physiologic* relief of IVC compression may occur prior to *anatomical* relief of IVC compression as other studies have shown an increase in maternal CO at 15° of left lateral tilt compared to supine (Lee et al., 2012).

The impact of posture on anatomy has physiological implications, and these physiological implications are more profound for some compared to others, with the former experiencing the symptoms and signs of SHS. Variations in individual anatomy from person to person have been attributed as the reason why every pregnant individual does not experience SHS and why those who experience it do so to different degrees. Several studies provide corroboration of physiologic changes secondary to anatomic alterations with improved maternal hemodynamic parameters in the left lateral posture compared to the supine or semi-Fowler’s posture (Jaffa et al., 2001; Kienzl et al., 2014; Ibrahim et al., 2021). To compensate for IVC compression and reduced IVC flow, other studies have added additional physiological insight demonstrating that the superficial, middle, and deep collateral venous pathways bypass the compressed and/or obstructed lumen and assist with return of blood to the right side of the heart (Humphries et al., 2017). The azygos system is particularly involved in this, which was demonstrated in an MRI study of participants in the late third trimester–there was a reduction in IVC blood flow by 85.3% at its origin and by 44.4% at the level of the renal veins in the supine posture compared with left lateral posture, with a concomitant increase in azygos vein blood flow by 220% (Humphries et al., 2019). However, in women with SHS, when supine, the compensatory increase in azygos flow is significantly blunted compared to those who do not have SHS (Humphries et al., 2020), indicating that reduced ability of the azygos system to bypass the occluded IVC may be a defining anatomical feature in SHS. In another study, these investigators also found that there are certain alterations in the collateral pathways, e.g., the ascending lumbar vein, that may make collateral bypass less effective (Humphries et al., 2017). One study noted that the extradural venous plexus is enlarged in the supine posture in late pregnancy, returning to normal pre-pregnant size in the left lateral posture (Hirabayashi et al., 1997), which we discuss further in the Spine section in Supplementary Material S2 – Musculoskeletal Maternal Posture-Physiology Interactions. Humphries *et al.* describe no significant difference in blood flow at the origin of the IVC between right- and left-lateral postures with 60° of tilt. However, they found a 35% reduction in IVC blood flow superiorly at the level of the renal veins in the right lateral posture compared to the left lateral posture (Humphries et al., 2019). On the arterial side, abdominal aorta blood flow does not decrease (when supine compared to left lateral) at the level of the renal veins but has been shown to be reduced by 32.3% at the level of the aortic bifurcation (Humphries et al., 2019).

While the scope of this review is limited to the resting state, for the purposes of understanding SHS further, we felt it helpful to include labor. There is some controversy in the literature of whether SHS can occur during labor, but consensus indicates that SHS would only occur during labor in a patient who is extremely sensitive to changes in intravascular volume. Indeed, there is one report of SHS occurring in labor in a patient with a stenotic aortic lesion who was being induced for cardiac decompensation (Baird and Arkoosh, 2012). A sinusoidal maternal heart rate pattern in this patient while in the supine position that correlated with her uterine contractions suggested cyclical variations in preload. That is, increased preload from autotransfusion of approximately 400 mL of blood into maternal venous circulation with each uterine contraction was followed by decreased preload as the gravid uterus relaxed between contractions, compressed the IVC again, obstructing venous return to the right side of the heart. This patient’s sinusoidal maternal heart rate resolved with a 400 mL intravenous crystalloid bolus and orienting this patient with left lateral uterine displacement. Apart from such patients, SHS does not occur in labor due to a variety of factors. First, engagement of the fetal head in the pelvis lower than the level of the sacral promontory is likely to relieve IVC compression. Second, SHS requires a relaxed uterus (Park and Hidaka, 1991), whereas strong uterine contractions during labor lift the uterus off the vertebral column, providing some relief of IVC compression. Third, autotransfusion of approximately 400 mL of blood from the relaxed uterus back into maternal venous circulation with each contraction combats SHS by increasing preload. Finally, it takes between three and 7 minutes for SHS to develop, but since contractions occur more frequently than this in labor, SHS does not have time to develop (Schneider et al., 1993).

##### Autonomic

Classically, the autonomic nervous system (ANS) is composed of a sympathetic branch (“fight or flight”) and a parasympathetic branch (“rest and digest”). These two opposing branches provide regulatory input into the cardiovascular system–sympathetic tone causing the HR to increase and parasympathetic tone causing it to decrease. When either of these branches are predominant over the other, the result is a reduction in HR variability (HRV), whereas HRV is increased when both components are actively opposing each other. In normal pregnancy, there is a rearrangement of the ANS described both as a shift toward parasympathetic predominance or as the consequence of an attenuated baroreflex (Speranza et al., 1998; Heiskanen et al., 2008).

Taking gestational age and posture into account, however, has shown that this rearrangement is more nuanced. Normal pregnancy results in *biphasic* changes in ANS activity. In the first trimester, the ANS shifts toward a lower sympathetic and higher parasympathetic modulation (Kuo et al., 2000). Relevant to the theme of this review, even in early pregnancy, the supine posture seems to affect indices of ANS balance (DSilva et al., 2014; Carpenter et al., 2015).

As gestation advances, however, the ANS shifts toward a higher sympathetic and lower parasympathetic tone (Blake et al., 1979; Ekholm et al., 1993; Kuo et al., 2000; Corrêa et al., 2019), especially in supine posture, (DSilva et al., 2014; Kuo et al., 2000; Blake et al., 1979). This biphasic change in ANS activity in pregnancy may be a result of the competing influences and resultant hemodynamic effects of physiologic volume expansion in the second trimester *versus* aortocaval compression from the enlarging gravid uterus in the third trimester (DSilva et al., 2014; Kuo et al., 2000; Maser et al., 2014). In late pregnancy, careful experiments have demonstrated that this shift in ANS tone is a state of decreased parasympathetic activity rather than increased sympathetic activity (Blake et al., 1979). Reduced parasympathetic activity at term is likely responsible for increased HR and CO (Ekholm et al., 1993; Heiskanen et al., 2008), whereas concomitant sympathetic predominance likely ensures stable peripheral vascular resistance and adequate maternal-side placenta supply (Heiskanen et al., 2008). Some authors have suggested that resting in lateral postures may provide more benefit to those who have higher sympathetic and lower parasympathetic tone when supine (Kuo et al., 2000). However, this may be true for all pregnancies–after 33 weeks’ gestation, left lateral posture has significantly lower sympathetic and higher parasympathetic tone compared to supine posture (Speranza et al., 1998).

The usual response to orthostatic stress (shifting from supine to standing) is one of sympathetic predominance to ensure BP is maintained via the baroreceptor reflex. With advancing gestation, however, supine posture is already a state of sympathetic predominance, meaning the usual ANS response to orthostatic stress (increase in sympathetic tone) is attenuated (DSilva et al., 2014), and this attenuation seems more pronounced in pregnancies affected by preeclampsia (Dyer et al., 2004; Rang et al., 2004). This may be because pregnancies affected by preeclampsia experience additional ANS imbalance, characterized by increased resting sympathetic tone as gestation advances, compared to their normotensive counterparts (Lewinsky and Riskin-Mashiah, 1998; Rang et al., 2004; Swansburg et al., 2005). This ANS imbalance is detectable both prior to pregnancy and as early as 8 weeks gestation in individuals who will go on to develop preeclampsia and is compounded by decreased circulating volumes in these patients (Rang et al., 2004). Other studies have proposed that observation of aberrant responses to orthostatic stress (due to this ANS imbalance) could be used as a clinical marker for preeclampsia (Ahmad et al., 1996; Staelens et al., 2015). Furthermore, studies of cardiovascular reflex testing (shifting from left-lateral to supine posture) have demonstrated sympathetic overreactivity in pregnancies with preeclampsia in comparison to normotensive pregnancies and non-pregnant controls (Lewinsky and Riskin-Mashiah, 1998), while others have reported null findings (Ekholm et al., 1994).

In summary, the ANS plays a central role in maintaining homeostasis in the face of orthostatic stress. The ANS is rearranged in pregnancy, with competing forces of physiologic volume expansion and aortocaval compression by the gravid uterus, ultimately resulting in sympathetic predominance via parasympathetic attenuation in late pregnancy and at term. Sympathetic overactivity and intravascular volume contraction seen in preeclampsia make it an interesting area of study from the perspective of maternal posture-physiology interactions.

#### Peripheral vascular

**FIGURE 3:**
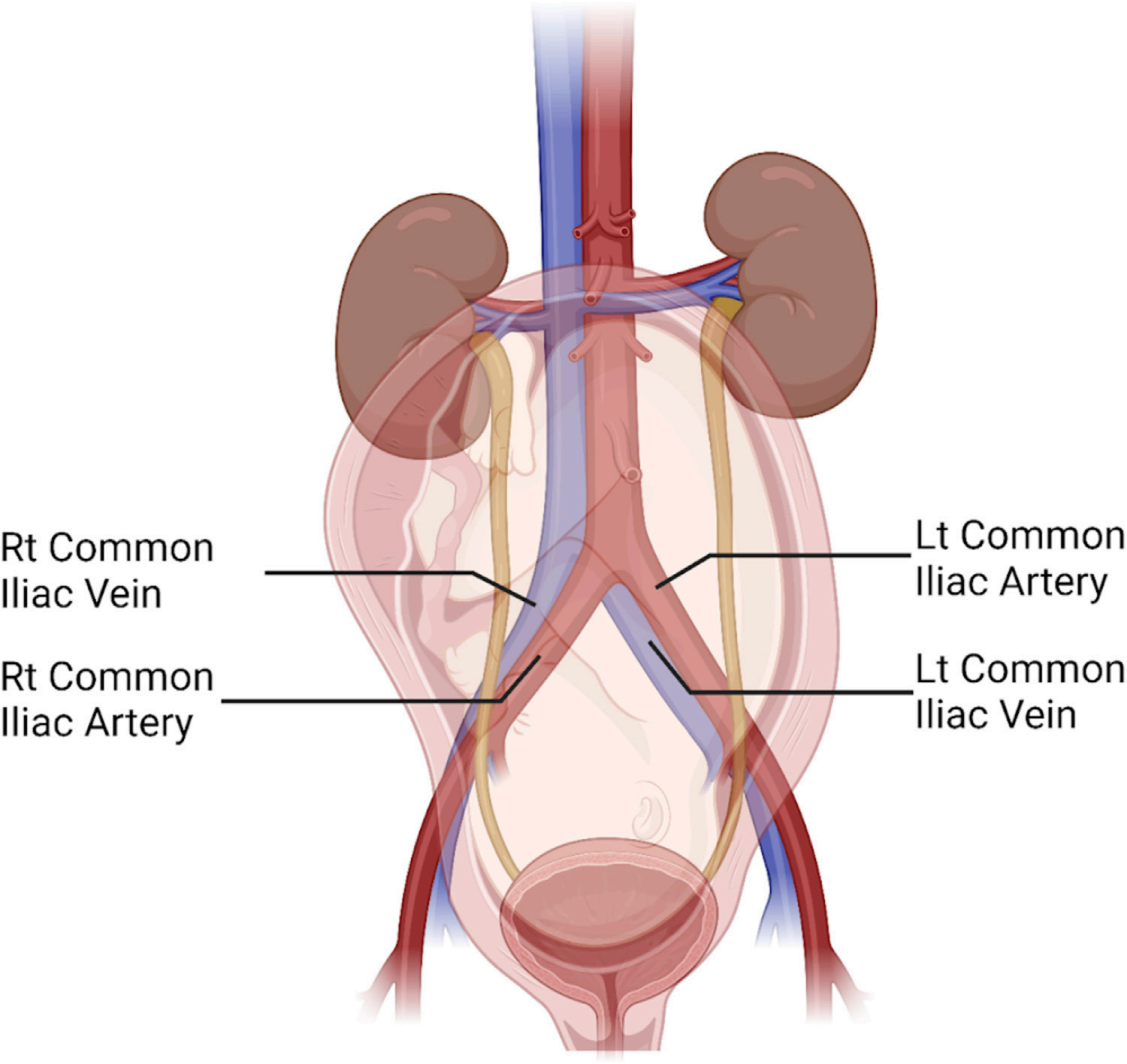
Illustration of the abdominopelvic region in the third trimester of pregnancy reveals the anterior-posterior orientation of the right common iliac artery crossing over the left common iliac vein, compressing it close to where it enters the inferior vena cava. The uterus and fetus are seen overlying the aortocaval bifurcation. Also shown are the left common iliac artery and the right common iliac vein. Figure created with Biorender.com.

Beyond cerebrovascular and cardiac physiology previously noted, the peripheral vascular system, particularly the venous side, is significantly impacted by gravitational forces with changes in posture. Invasive measurements of venous pressure demonstrate an increase with pregnancy, with the authors noting compression of the pelvic vessels in the standing posture (Runge, 1924; Schneider et al., 1993). As gestation progresses, this pressure increases further, doubling lower extremity (LE) venous pressure in the supine posture and quadrupling it in the standing posture (Runge, 1924; Schneider et al., 1993). It is unsurprising then that edema, varicose veins, and venous stasis are common during pregnancy, possibly contributing to deep vein thrombosis (DVT), or more seriously, pulmonary emboli (Chan et al., 2001). The left LE appears to be disparately impacted by DVT during pregnancy, with some studies reporting more than 80% of DVT’s occurring in the left LE (Ray and Chan, 1999; James et al., 2005). This could be a result of compression of the low-pressure, left common iliac vein near its entry into the IVC by the overlying, high-pressure, right common iliac artery and the overlying gravid uterus as shown in Figure 3 (Benninger and Delamarter, 2013; Devis and Knuttinen, 2017).

By the same token, risk of pulmonary emboli may be reduced during cesarean birth through maternal posture in reverse Trendelenburg where the patient in a supine posture has their head elevated slightly above their feet (Fong et al., 1991). Here, the authors postulate that a decrease in central venous pressure and venous return, likely a result of increased LE venous pressure and congestion, prevent dislodgement of a DVT in the LE and subsequent deposit of the emboli in the lungs (Fong et al., 1991). These venous pressure changes also have clinical consequences after pregnancy, where risk of non-saphenous venous reflux, which is implicated in venous stasis and DVT, increases with female sex and increasing number of gestations (Labropoulos et al., 2001).

Venous compression impacts other arterial-side vasculature as well. For example, uterine artery resistivity index increases with IVC compression in supine compared to the left lateral posture (Ryo et al., 1998). Interestingly, a small (<10th percentile) IVC diameter in the left lateral position could suggest gestational hypertension, although this was limited in its positive predictive value (Ryo et al., 1999). These suggest that maternal posture and the gravitational impact of the gravid uterus on the venous system may have serious implications beyond the LE’s.

With an understanding of these pressure changes and serious clinical impact, some studies assess the impact of hemodynamically protective postures on the vasculature, generally finding it assists with LE venous return (Palmgren and Kirkinen, 1996; Macklon et al., 1997; Duddy and McHugo, 2014). Specifically, the lateral positions decrease LE vein diameter, increase IVC diameter, and increase flow in the contralateral leg suggesting the LE venous system is no longer congested and impeded by the gravid uterus (Macklon et al., 1997; Ryo et al., 1999; Duddy and McHugo, 2014). A systematic review and meta-analysis of RCT’s of postural interventions for the prevention of LE DVT’s in the postpartum period in women with pregnancy-induced hypertension is currently underway (Chen et al., 2021).

#### Hepatic

Pregnancy is associated with a significant increase (>60%) in splanchnic blood flow that begins in early pregnancy and peaks at the end of the second trimester. Two-thirds of the blood flow in the splanchnic system is returned by the liver through the portal vein. Given that the splanchnic system is a major regulator of blood flow in response to hemodynamic stress (e.g., exercise, postural changes) via venoconstriction and shunting blood away from itself and toward vital organs, researchers have investigated portal vein blood flow across pregnancy to further elucidate the role of the splanchnic system relative to maternal-fetal hemodynamics (Clapp et al., 2000). Investigation of the splanchnic system is also motivated by the concept that its changes in blood flow reflect similar changes in uterine blood flow and, thus, potential fetoprotective mechanisms. Clapp *et al.* found that the venoconstrictive response to postural stress (changing from left lateral recumbent to standing) prior to pregnancy is attenuated in early and mid pregnancy and almost non-existent by late pregnancy, which indicates that the blunting of hormonal-induced venous reactivity seen in the peripheral circulation in pregnancy is also present in the splanchnic bed (Clapp et al., 2000). Despite less venoconstriction, however, they still found that changing from the left lateral recumbent to standing posture results in a significant reduction (25%–34%) in portal venous flow in early, mid, and late pregnancy.

Hepatic venous Doppler waveforms have been shown to undergo marked changes as pregnancy advances, from a mostly normal and minimally flat middle hepatic vein waveform between 12–20 weeks and progressing to a mostly flat and minimally normal waveform between 30–40 weeks (Roobottom et al., 1995). A study by Pekindil *et al.* demonstrated that these pregnancy-induced alterations in hepatic venous pulsatility (HVP) are partially normalized 2 days postpartum and do not completely return to normal in most patients until 6–8 weeks postpartum (Pekindil et al., 1999). While all examinations were completed in the maternal supine position, Pekindil et al. hypothesized that the marked pregnancy-induced changes in HVP are probably only minimally influenced by compression of the liver, iliac vessels, and IVC by the gravid uterus since the changes in HVP were still observed in 40% of their participants at 6–8 weeks postpartum despite universal uterine regression by this time (Pekindil et al., 1999). Other contributors of pregnancy-induced changes in HVP include a significant increase in the portal vein velocity and flow rate coupled with significant changes in cardiac output during pregnancy–the relative contribution of these effects remains open to debate (Roobottom et al., 1995; Pekindil et al., 1999).

### Respiratory

Approximately 20% of the adult human body’s 320 paired muscles are involved in breathing (Pilarski et al., 2019). Bearing testimony to the complexity of this vitally important physiology, some 63 muscles assisting breathing, most paired bilaterally, are under the control of a highly intricate neural circuitry largely housed in the medulla oblongata that automatically adjusts respiration to demand with little volition by or awareness to the individual (Pilarski et al., 2019). It is no surprise that, even aside from the physiologic demands of pregnancy, much about respiratory physiology remains poorly understood or not understood at all.

The effects of posture on the respiratory system during pregnancy have not been studied in depth until recently. One area of research focuses on how functional residual capacity (FRC) of the lungs changes with posture in pregnancy. It has been established that FRC decreases in pregnancy and is maximally reduced in the third trimester (Prodromakis et al., 2004; Hignett et al., 2011). To add to this, studies have shown that FRC is even lower in the supine posture due to the increase in abdominal pressure in this position. This increase in pressure causes the alveolar-arterial oxygen difference to increase (Prodromakis et al., 2004; Hignett et al., 2011) and worsens lung compliance (Aretha et al., 2017). Furthermore, Hignett *et al.* showed a significant increase in FRC from supine to the 30° head-up posture, which indicates that a large change in posture is not always necessary to improve FRC (Hignett et al., 2011). Using positioning to increase FRC in pregnancy can have important implications. In cases of apnea, hypopnea, or a situation where oxygen is rapidly depleted (e.g., induction of anesthesia), the FRC, if pre-oxygenated, functions as an oxygen reservoir. Increasing FRC may result in a significant increase in time to oxygen desaturation; however, while time to desaturation is significantly longer in the 45° head-up compared to supine posture in non-pregnant patients, evidence suggests that pregnant patients desaturate their blood of oxygen much faster than non-pregnant patients, and there is no difference in time to desaturation between these postures in term pregnant patients (Baraka et al., 1992).

In the setting of reduced FRC, as seen in pregnancy, increased ventilation-perfusion mismatching and hyperventilation can lead to an increase in intrapulmonary shunting. One study examined the amount of shunting in normotensive women in different postures at 36–38 weeks using pulmonary artery and radial artery canalization (Hankins GDV. et al., 1996). This report found that intrapulmonary shunting was significantly higher in the right lateral posture (15.2%) compared to the knee-chest posture (12.8%; definition for “knee-chest” was not given) and standing posture (13.0%). However, this was a small study (n = 10), and more research should be done to further examine the differences in shunting between different postures.

During sleep, pregnant individuals experience more respiratory events and more and deeper oxygen desaturations when supine regardless of obstructive sleep apnea (OSA) status (Dunietz et al., 2020). This is corroborated by a study by Wilson *et al.* (higher apnea-hypopnea index [AHI], 3% oxygen desaturation index, and respiratory disturbance index when supine), which was published during the writing of this review and included here for context (Wilson et al., 2022). In the first trimester, there is no difference between the arterial partial pressure of oxygen (PaO_2_) in the supine and sitting postures (Spiropoulos et al., 2004); however, in the second and third trimesters, PaO_2_ is significantly lower in the supine posture compared to the sitting posture (Prodromakis et al., 2004; Spiropoulos et al., 2004), and supine PaO_2_ is significantly lower during the third trimester than in the postpartum period (Trakada et al., 2003). Only one report contradicts the notion that PaO_2_ is affected by maternal posture, but this study was small (n = 10) and conducted at moderate altitude (1388 m) (Hankins GD. et al., 1996).

In addition to PaO_2_, the effects of posture on oxygen saturation (SpO_2_) and respiration rate (RR) have also been studied. However, studies differ on whether posture can cause a significant change in either of these metrics. One report examining the effects of posture on healthy women between 20–37 weeks’ gestation found a significant decrease in RR in the prone posture compared to the left lateral posture with no significant change in SpO_2_ (Oliveira et al., 2017). The study also found that SpO_2_ was increased significantly in prone posture compared to Fowler’s posture (sitting) and supine posture. Other studies show no significant differences in SpO_2_ or RR between different postures. One such study by Dennis *et al.* examining healthy and preeclamptic women at term showed no significant changes in SpO_2_ or RR in the prone or lateral posture for either the normotensive or preeclamptic women (Dennis et al., 2018a). A third study focusing on healthy women at term found no significant differences in SpO_2_ or RR in the left lateral decubitus, ramped posture with wedge, or ramped posture alone (Dennis et al., 2018b). One important thing to note is that PaO_2_, discussed in the previous paragraph, measures the partial pressure of oxygen in arterial blood (invasive), while SpO_2_ measures the percent saturation of hemoglobin with oxygen (non-invasive). At high SpO_2_ levels (above 90%), there can be a significant change in PaO_2_ without much change in SpO2, which could explain why most studies focusing on PaO_2_ show a change in PaO_2_ with position changes in pregnancy, while studies focusing on SpO_2_ had differing results.

Clinically, in the postpartum period, sleeping with the upper body elevated at 45° can significantly improve OSA (as measured by AHI) compared to the non-elevated posture without affecting sleep quality or quantity. This is partially explained by an increased upper airway diameter in the elevated posture (Jung et al., 2014; Zaremba et al., 2015). Sleeping with the upper body elevated 45° in the postpartum period has been shown to successfully treat OSA in half of those with moderate to severe OSA (Zaremba et al., 2015).

Another subject of research is the relationship between airway size and maternal posture. It has been shown that airway size decreases as gestation advances, but studies conflict on whether there is a significant difference in airway size between different body postures. According to Izci and colleagues, pregnant individuals have significantly wider upper airways than non-pregnant counterparts when seated. However, there was no difference between pregnant and non-pregnant patients in the supine posture. When pregnancies with preeclampsia were considered, they had a narrower pharynx at the oropharyngeal junction compared to pregnancies without preeclampsia and non-pregnant controls in both the seated and supine postures (Izci et al., 2003). On the contrary, a later study showed that while there is a significant difference between airway size in pregnant individuals and non-pregnant controls, there was no significant difference in size between postures (Izci et al., 2006).

In addition to FRC and airway size, Harirah *et al.* found that peak expiratory flow rate (PEFR) in pregnancy differs according to posture. While PEFR is known to decrease throughout pregnancy, they found that it decreases significantly faster in the supine posture as opposed to standing and sitting (Harirah et al., 2005). While significant progress has been made in understanding changes in the respiratory system during pregnancy, there is certainly more work needed on the impact of maternal posture.

In summary, supine posture in pregnancy presents an additional stressor to an already-challenged respiratory system. Indeed, in one study of myasthenia gravis (MG) in pregnancy, three of seventeen MG patients studied over a 22-year period in a single center presented with symptoms, but they did not yet have a diagnosis at delivery. Of these, one presented with discrete supine dyspnea and subsequently developed severe dyspnea at time of cesarean section under spinal anesthesia, which required mechanical ventilation and ICU admission (Almeida et al., 2010). These respiratory symptoms and complications, precipitating in the supine position, further demonstrate the effect that maternal posture can have on respiratory physiology in pregnancy.

### Renal

As the renal system helps regulate blood pressure, fluid balance, and more, postural changes during pregnancy may provide clues for therapeutic postures or physiologic mechanisms of disease. The renin-angiotensin-aldosterone system (RAAS) is key here, and most studies evaluating postural changes look at hormones which impact this process. In this section, we consider interactions between maternal posture and renal hormones, renal function, and renal hemodynamics.

Atrial natriuretic peptide (ANP) is one such hormone and is released in response to atrial stretch to decrease renin, angiotensin II, and aldosterone production, decreasing blood pressure. With upright posture, ANP decreases (Lowe et al., 1991a; Poulsen et al., 1995). With lateral recumbency, ANP increases (Lowe et al., 1991a; Poulsen et al., 1995; Almeida et al., 2009). This may be altered in hypertensive disorders of pregnancy (HDP) with one study reporting an overall elevation of ANP in pregnancy-induced hypertension with a similar response to postural stimuli (Lowe et al., 1991b), while another reports no significant difference in baseline ANP and no significant differences with a head-down tilt (Poulsen et al., 1995). This may be a result of different experimental methods, where a larger increase in blood volume is returned to the heart upon changing from an upright position to the lateral recumbent position (Lowe et al., 1991a; Lowe et al., 1991b), while a shift from lateral recumbency to a head down tilt may not be as great of a change in venous return (Poulsen et al., 1995).

Renin and aldosterone production is also altered with changes in posture. Plasma renin is reported to decrease in the left lateral recumbent posture in two studies reported by Fagundes et al. and Almeida et al., while Poulsen reports no significant change with a head-down tilt (Fagundes et al., 1992; Poulsen et al., 1995; Almeida et al., 2009). Others report an increase in plasma renin in the upright posture (Bentley-Lewis et al., 2005). With a decrease in plasma renin, it follows that aldosterone should also decrease. This is the case during normal pregnancy, where a left lateral recumbent posture decreases aldosterone and an upright posture increases aldosterone, but overall correlation between plasma renin and serum aldosterone levels is low during normal pregnancy (Fagundes et al., 1992; Bentley-Lewis et al., 2005; Almeida et al., 2009). Conversely, in HDP there is a high correlation between plasma renin and serum aldosterone levels with a change in posture (Fagundes et al., 1992), suggesting that the desensitization of the RAAS, which happens during normal pregnancy, is altered in these conditions.

Lastly, creatinine clearance, a basic metric of renal health, also changes with a change in posture. Serum creatinine appears to decrease in the upright position and increase in the left lateral position (Lohsiriwat and Imrittha, 2008; Almeida et al., 2009), which is likely a dilution-concentration effect and is corroborated by increased sodium excretion and diuresis in the left lateral posture (Almeida et al., 2009). This is consistent with our previous discussion of ANP, a strong diuretic, which increases in the left lateral posture and decreases in the upright posture.

It is clear that the kidneys are not immune to changes in posture during pregnancy. Indeed, some studies even report changes in blood pressure with a shift in posture, with the lowest blood pressure being reached in the left lateral recumbent position (Fagundes et al., 1992; Bentley-Lewis et al., 2005; Almeida et al., 2009). Recent work has implicated supine posture after 24 weeks in the development of hypertension and preeclampsia (Tokunaga et al., 2000; Qureshi et al., 2019). At this time point, the fundus of the gravid uterus reaches the level of the renal veins, and the left renal vein at the aortic crossway is particularly vulnerable to compression in a manner similar to what is seen in nutcracker syndrome (Reuter et al., 2016). The majority of patients have alternate pathways for venous return to the right atrium (such as through the hemiazygos vein), but up to 25% of patients may not have adequate collaterals (Beckmann and Abrams, 1980; Li et al., 2011). Without alternative drainage, acute renal venous outflow obstruction occurs, leading to an increase in intrarenal pressure resulting in decreased arterial perfusion and a condition akin to a renal compartment syndrome. This activates renin-mediated hypertension initially, and with repeated injury, ischemia-mediated hypertension, which may have implications in preeclampsia development (Tokunaga et al., 2000; Reuter et al., 2016; Qureshi et al., 2019). Depending on severity, this may also impact the left ovarian vein and placental drainage. Related to this physiology, we find the example of quadruped pregnancies intriguing: they do not experience compression of the retroperitoneal vessels by the gravid uterus, nor do they experience hypertension associated with pregnancy. We return to this unique comparison in the Abdominal section.

Varied results have been reported with the use of the “roll-over test” in which blood pressure is measured in the supine then left lateral posture as a predictive tool for HDP, where a difference in diastolic pressure greater than 20 mmHg indicates a higher risk for preeclampsia development (Gant et al., 1974). Some authors have reported poor negative predictive value with this test but typically with use at a single time point only, and serial use (especially as pregnancy progresses, when the renal vessels are more prone to compression) may improve test sensitivity (Okonofua et al., 1991; Reuter et al., 2016). As HDP impact 2%–8% of pregnancies (Duley, 2009), more research is warranted vis à vis the potential therapeutic impact of lateral recumbent posture during pregnancy and the diagnostic value of the roll-over test.

### Genitourinary

Urinary retention, calyceal dilation, and hydronephrosis are known to commonly occur in pregnancy, with the latter two becoming more common as pregnancy progresses. Strategic posturing may assist in all of these issues. Urinary retention with a retroverted gravid uterus was reported in a few case studies in which the uterus displaced the cervix anteriorly, compressing the urethra and preventing emptying of the bladder (Love and Howell, 2000; Yang and Huang, 2002; Yang and Huang, 2004). Manual reduction of this is possible and may occur spontaneously. Yang and Huang recommend prone positioning or a standing position to assist with relieving this issue (Yang and Huang, 2004). Yang *et al.* reported that standing or squatting does not increase post-void residual, but rather significantly decreases it, suggesting that voiding in the standing or squatting position may have advantages (Yang et al., 2017).

Beyond urinary retention and challenges with emptying the bladder during pregnancy, renal pelvis dilation and hydronephrosis are common and it can be difficult to distinguish between ureteral obstruction from a stone *versus* the gravid uterus. Color Doppler evaluation for the presence of ureteral jets can be a helpful tool, but this approach can be misleading if the jets are not seen. Use of contralateral positioning may alleviate pressure from the gravid uterus on the affected ureter, allowing visualization of the missing ureteral jet (Wachsberg, 1998; Karabulut and Karabulut, 2002). Here, again, it is clear that lateral recumbent positioning where the weight of the gravid uterus is removed or relieved from the internal organs (bladder, kidneys, ureter) may be helpful, suggesting that clinicians may consider this as a possible therapeutic position in instances of ureteral obstruction or urinary retention.

### Musculoskeletal

The musculoskeletal system performs several vital functions. One vital function of the musculoskeletal system is to withstand gravitational force, and at the same time, gravitational force is critical for the development and maintenance of the musculoskeletal system. Hence, pregnancy-induced changes in mass and moment arms portend posture-physiology interactions between pregnancy and the musculoskeletal system from head to toe. These are discussed separately in Supplementary Material S2 – Musculoskeletal Maternal Posture-Physiology Interactions.

### Gastrointestinal

Despite constipation and gas pain being common in pregnancy and a common topic of pregnancy blogs and medical websites, our systematic search did not find any publications related to these topics and the effects of posture or gravity in pregnancy, which is an area of opportunity for future work. In patients with gastroesophageal reflux disease (GERD), head of the bed elevation and left lateral decubitus position improves the overall time that the esophageal pH is less than 4.0 (Kaltenbach et al., 2006). Head of bed elevation, therefore, is considered an effective lifestyle intervention for nighttime GERD symptoms (Katz et al., 2022). Indeed, in the second and third trimester of pregnancy, a 2 week trial of positional therapy to keep the upper body inclined during sleep has been reported to reduce symptoms in patients with frequent moderate to severe nocturnal GERD (Kichler et al., 2015).

Pregnancies with hyperemesis gravidarum report greater perceived balance instability during standing and, compared to pregnancies without this condition, attempt to stabilize their bodies by reducing overall body sway (Yu et al., 2013). This reduced postural sway may be a deliberate, albeit unconscious, effort to increase stability and reduce symptoms (Yu et al., 2013). In the first trimester, Johnson *et al.* assessed the potential for changes in pulse rate and blood pressure from supine to standing after 5 minutes each position to determine hydration adequacy in patients with hyperemesis gravidarum (Johnson et al., 1995). The increase in HR from supine to standing seen pre-administration of fluids was attenuated post-administration. Similarly, the decrease in SBP from supine to standing seen pre-administration of fluids was attenuated post-administration and the authors concluded that while the assessment was not sensitive enough to be used to screen for hypovolemia, it could still provide supplemental clinical information on hydration progression (Johnson et al., 1995).

### Abdominal

It is well established in the critical care community that posture affects intra-abdominal pressure (IAP) – in particular, as head of bed (HOB) elevation increases, IAP undergoes a clinically significant increase (Cheatham et al., 2009; Yi et al., 2012). This may be concerning given that 30°–45° HOB elevation is by far, in the authors’ experience, the most common daytime resting posture of patients admitted to high-risk antenatal units. We submit an evaluation of the safety of reclining in the 30°–45° HOB elevated posture in late pregnancy as an area of future research. While acutely increased IAP is pathological (i.e., intra-abdominal hypertension [IAH] is defined as IAP ≥12 mmHg, and abdominal compartment syndrome is defined as IAP ≥20 mmHg) (Kirkpatrick et al., 2013), normal pregnancy induces a temporary increase in IAP, and this is understandable through anatomical study. In humans, the abdominal cavity is relatively smaller and the pelvic cavity is relatively larger than in non-human animals (Abitbol, 1993). As a result, several organs that are mostly intraabdominal in non-human animals are mostly pelvic in humans (e.g., bladder, genital organs, and rectosigmoid colon). In non-human mammals and non-human primates, the pregnancy starts and remains in the abdominal cavity until term, whereas in humans, the pregnancy is contained within the pelvic cavity within the first trimester and expands into the abdominal cavity in the second and third trimesters. A major difference in human pregnancy is that the human uterus and fetus are relatively large compared to the relatively small abdominal cavity – the volume of the human uterus at term is 1.57 times larger than the abdominal cavity (Abitbol, 1993). Whereas the anterior abdominal wall acts as a “hammock” on which the uterus rests in quadrupeds, upright posture of humans along with the anterior abdominal wall fascia and musculature pull the uterus posteriorly compressing it against the lordotic lumbar vertebral column and retroperitoneum.

Increased IAP has been recently theorized as participating in preeclampsia pathogenesis via hypertension, secondary renal injury, and proteinuria in both normal pregnancies and pathological pregnancies (e.g., hydatidiform mole where rapid increase in IAP occurs) (Zhang, 2015). We found little original research studying the effect of maternal position on IAP in the antepartum period. In term, non-laboring patients awaiting elective CS, the supine IAP is significantly higher than the lateral tilt (10°) IAP, and the IAP is in the range of IAH for more than a 25% of patients in both positions (but a greater percentage of patients in the supine position) (Chun et al., 2012; Garg et al., 2021). In neither position, however, is IAP associated with organ dysfunction (Garg et al., 2021). IAP’s in the IAH range were significantly correlated with obesity and preeclampsia in both positions (Garg et al., 2021). Postpartum, the IAP declines immediately (Al-Khan et al., 2011; Ozkan et al., 2015). Following cesarean section, IAP is significantly lower in the supine posture compared with semirecumbent, and IAP significantly correlates with BMI and arterial hypertension and is in the IAH range in the latter group (Abdel-Razeq et al., 2010).

### Endocrine

Maternal posture may affect neurohormone release and clearance, which could be manifested by alterations in blood pressure. In a study between 36–40 weeks gestation with and without preeclampsia compared to non-pregnant controls, Kaaja *et al.* showed that SBP after position change from left lateral (measured 30 min after assuming the position) to supine position (measured 10 min after assuming the position) is significantly reduced in pregnancies with preeclampsia and significantly increased in normotensive pregnancies (Kaaja et al., 2004). Return to the left lateral position again resulted in the opposite effect (increased SBP in pregnancies with preeclampsia and decreased SBP in normotensive pregnancies), which remained statistically significant. They also demonstrated that plasma-renin activity and norepinephrine levels in pregnancies with preeclampsia were significantly higher with upright position (measured 10 min after assuming the position; compared to left lateral position and supine position) than in normotensive pregnancies. The norepinephrine level also remained higher in pregnancies with preeclampsia compared to normotensive pregnancies after resumption of the left lateral position (measured 30 min after assuming the position) (Kaaja et al., 2004). While higher levels of norepinephrine may indicate sympathetic overactivity, the authors noted that delayed clearance of norepinephrine from plasma may also contribute to the observed effect. Changes in other hormones (e.g., ANP, BNP, epinephrine, and endothelin-1) with position were not different between pregnancies with preeclampsia, normotensive pregnancies, and non-pregnant controls.

A follow up study five to 6 years following pregnancy found that individuals with previous preeclampsia had sustained higher levels of norepinephrine at rest compared with those without a history of preeclampsia, and they had a positive correlation between endothelin-1 levels and SBP and DBP in the upright position, which was not seen in with those without a history of preeclampsia (Lampinen et al., 2014).

### Skin

Maternal posture may affect the skin. Striae gravidarum (SG), also known as “stretch marks”, is a condition that manifests around 6–7 months gestation and is characterized by pink linear patches on the skin that generally localize to the abdomen, breasts, and thighs. Particular to maternal position and gravity interactions, a recent study by Ren and colleagues found that SG were associated with significantly lower height and higher pre- and post-pregnancy BMI (Ren et al., 2019). There was a trend of decreased daily sitting time, increased gestational weight gain, and increased infant birth weight with SG, but these were not statistically significant. Within pregnancies with SG, abdominal region SG was significantly associated with non-sedentary behavior (sitting time less than 5.9 h per day) and was significantly more severe (more than half of the body region covered with SG) with non-sedentary behavior (Ren et al., 2019). Leg SG was associated with sedentary behavior (sitting time 5.9 h per day or more) and was significantly more severe with sedentary behavior (Ren et al., 2019). Hip SG occurrence and severity, however, was not significantly associated with activity level. To our knowledge, these findings are the first to show an association between maternal posture and SG in the antepartum period.

## Discussion

**FIGURE 4:**
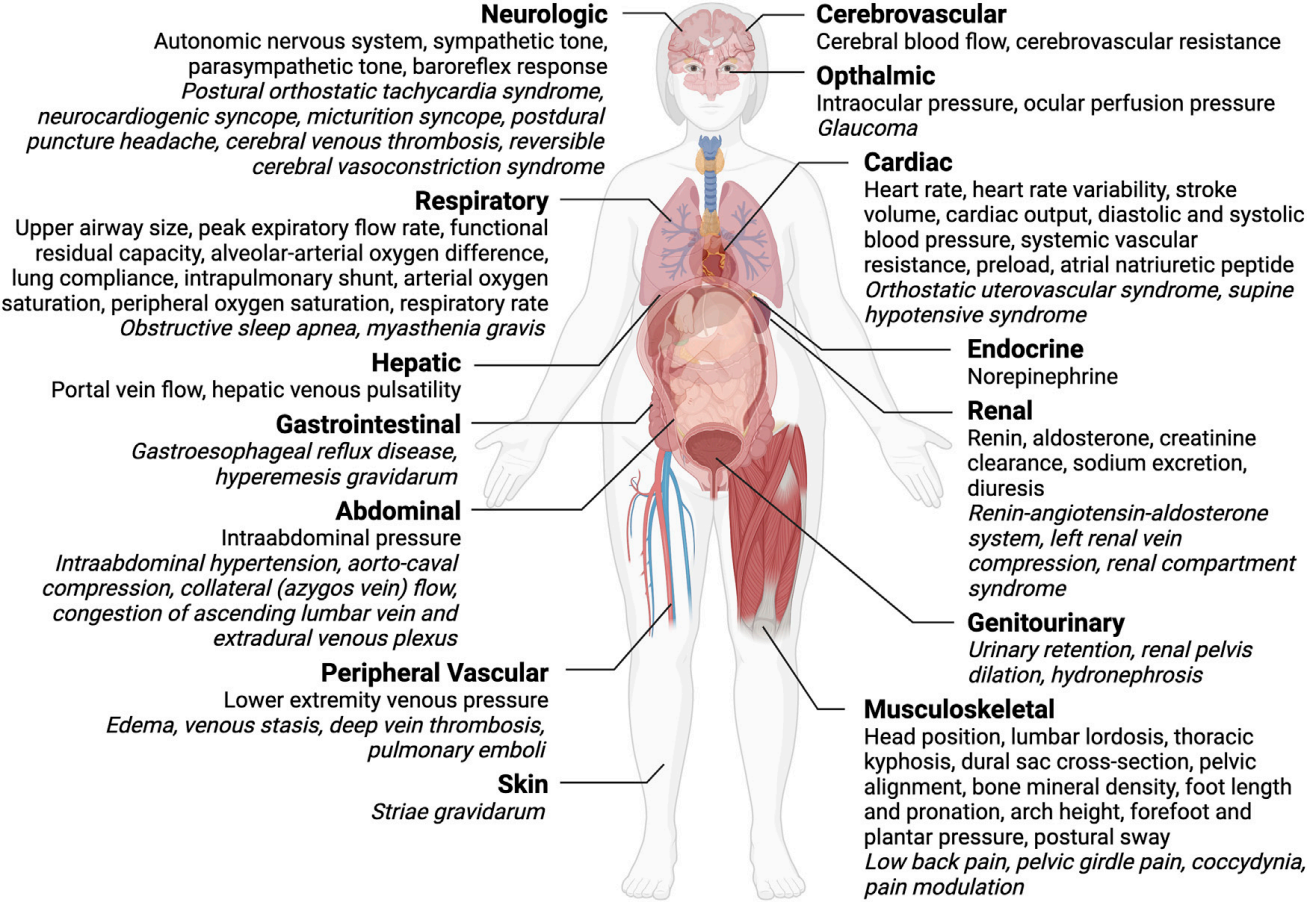
Summary diagram of maternal posture-physiology interactions during pregnancy grouped by organ system (bold text). Physiologic variables affected by maternal posture are in plain text, while pathophysiology is in italic text. Figure created with BioRender.com.

This review of maternal posture-physiology interactions stemmed from a common understanding within the broader medical community that gravity and posture interact with the human body in a manner that may impact both function and disease. Indeed, our review encountered numerous studies of such physiology and conditions occurring within the context of pregnancy (see Figure 4), including POTS, (Glatter et al., 2005; McEvoy et al., 2007) neurocardiogenic and micturition syncope, (Huang et al., 2003; Sherer et al., 2005) PDPH, (Buddeberg et al., 2019) reversible cerebral vasoconstriction syndrome, (Lenger and Barrier, 2014) glaucoma, (Kara et al., 2013) DVT, (Benninger and Delamarter, 2013; Devis and Knuttinen, 2017) OSA, (Dunietz et al., 2020) MG, (Almeida et al., 2010) and GERD, (Kichler et al., 2015) to name a few. Against the backdrop of such conditions, the challenge of pregnancy to homeostasis was usually found to exacerbate the condition, although in the case of POTS, the condition is initially alleviated by pregnancy and then exacerbated with advancing gestation (Glatter et al., 2005; McEvoy et al., 2007). In some cases (e.g., MG), it was the postural stresses unique to pregnancy (i.e., supine dyspnea) that prompted the diagnostic workup for the condition (Almeida et al., 2010). We concur that posture-physiology/pathophysiology relationships in pregnancy are complex and contain various layers and directions of interactions; for example, with GERD, pregnancy-induced elevations of serum progesterone relax lower esophageal sphincter tone, exacerbating reflux, as does the growing gravid uterus pushing on the stomach from below, especially so when supine. The impact that posture has on posture-sensitive conditions in the setting of pregnancy is certainly an interesting avenue of future research. In Table 2, we provide a summary of the impact, on maternal physiology, of the five main maternal postures uncovered in our review (supine, left lateral, right lateral, sitting, and upright) grouped by organ system. In Table 3, we give a summary of the impact of the five main maternal postures on various pathophysiological entities, again grouped by organ system.

**TABLE 2:** Summary table of the impact of maternal posture on physiology during pregnancy grouped by organ system for five main maternal postures.

	Supine	Left lateral	Right lateral	Sitting	Upright
Neurologic & Autonomic					
Parasympathetic tone^a^	Ref	↑			↑
Sympathetic tone^a^ ^,^ ^b^	Ref	↓			↓
Ophthalmic					
Intraocular pressure	Ref	NS	NS	↓	
Ocular perfusion pressure	Ref	↓	↓	↑	
Cerebrovascular					
Cerebral blood flow velocity	↓^c^	Ref		↑^c^ ↓^e^ NS^f^	
Cerebrovascular resistance	↑↑^c^ ↑^g^	Ref			
Cerebral blood flow volume	↓^h^			Ref	
ICA blood flow velocity	↓^h^			Ref	
Cardiovascular					
See Table 1					
Peripheral vascular					
Lower extremity venous pressure	↑	Ref			↑↑
Uterine artery resistivity index	↑	Ref			
Inferior vena cava diameter	↓	Ref			
Lower extremity vein diameter	↑	Ref			
Hepatic					
Splanchnic vasoconstriction		Ref			↑^i^
Portal venous blood flow		Ref			↓
Respiratory					
Functional residual capacity	↓				Ref
AHI, ODI-3, RDI	↑	Ref			
Arterial partial pressure of O_2_	↓^j^			Ref	
Upper airway diameter	Ref				↑
Peak expiratory flow rate	↓			Ref	
Renal					
Serum atrial natriuretic peptide	Ref				↓^i^
Serum renin		Ref			↑
Serum aldosterone		Ref			↑
Serum creatinine		↑			Ref
Sodium excretion		↑			Ref
Diuresis		↑			Ref
Musculoskeletal					
Dural sac					
Axial cross-sectional area	Ref	↑	↑		
Transverse maximum diameter	Ref	↑	↑		
Shape	Circular	Oval	Oval		
Epidural venous plexus	Engorged	Shrunk	Shrunk		
Abdominal					
Intra-abdominal pressure	↑	Ref^k^			
Endocrine					
Serum norepinephrine	Ref	Ref			↑^g^ ↑↑^c^

Abbreviations: Ref indicates reference posture. NS, indicates non-significant difference; ICA, indicates internal carotid artery; AHI, indicated apnea-hypopnea index; ODI-3, indicates 3% oxygen desaturation index. RDI, indicates respiratory disturbance index. O_2_ indicates oxygen.

^a^ In late pregnancy.

^b^ This seems contrary to the usual baroflex response (i.e., increase in sympathetic tone upon standing from supine); however, gestation advances, the baroreflex response is attenuated because the supine posture is already a state of sympathetic predominance, and this attenuation is especially pronounced in pregnancies with preeclampsia.

^c^ In pregnancies with preeclampsia.

^d^ In pregnancies with severe preeclampsia.

^e^ In pregnancies with mild preeclampsia and GH.

^f^ In pregnancies with chronic hypertension.

^g^ In normotensive pregnancies.

^h^ In pregnancies with supine hypotensive syndrome.

^i^ Attenuated as gestation advances.

^j^ In the second and third trimesters.

^k^ Reference is supine with 10° lateral tilt; higher intra-abdominal pressures seen in pregnancies affected by obesity and pregnancies with preeclampsia.

**TABLE 3:** Summary table of the impact of maternal posture on various pathophysiologic entities during pregnancy grouped by organ system for five main maternal postures.

	Supine	Left lateral	Right lateral	Sitting	Upright
Neurologic					
POTS symptoms	Ref				↑
Neurogenic syncope	Ref			↑	
Micturition syncope	Ref			↑	
PDPH headache	Ref			↑	↑
CVT headache	Ref			↑	↑
RCVS headache	Ref			↓	↓
Circulatory system					
SHS symptoms	↑	Ref		Ref	
Lower extremity venous stasis	↑	Ref = lateral postures			↑↑
Respiratory					
Obstructive sleep apnea	↑	Ref = lateral postures			
Myasthenia gravis	↑			Ref	
Renal					
Left renal vein compression^a^	↑	Ref			
Musculoskeletal					
Low back pain		Ref = lateral postures		↑	↑
Pelvic girdle pain	Ref = horizontal postures			↑	↑↑
Gastrointestinal					
GERD symptoms	Ref = horizontal postures			↓	
Skin					
Striae gravidarum				Leg^b^	Abdominal^c^

Abbreviations: POTS, indicates postural orthostatic tachycardia syndrome; PDPH, indicates postdural puncture headache; CVT, indicates cerebral venous thrombosis; RCVS, indicates reversible cerebral vasoconstriction syndrome; SHS, indicates supine hypotensive syndrome; GERD, indicates gastroesophageal reflux disease.

^a^ After 24 weeks’ gestation.

^b^ Sedentary behavior (sitting time 5.9 h per day or more).

^c^ Non-sedentary behavior (sitting time less than 5.9 h per day).

We also found literature that suggests posture interacts with comorbidities in pregnancy, such as obesity and hypertension (Ekholm et al., 1994; Carson et al., 2002), and with conditions specific to pregnancy, including preeclampsia (Kanayama et al., 1997; Chipchase et al., 2003; Swansburg et al., 2005; Dennis et al., 2018a), gestational hypertension (Ekholm et al., 1994; Ryo et al., 1999), SHS (Ikeda et al., 1992; Humphries et al., 2020), incarcerated retroflexed gravid uterus (urinary retention) (Yang and Huang, 2004), hyperemesis gravidarum (Johnson et al., 1995), and striae gravidarum (Ren et al., 2019). We suggest that the work elucidating such interactions should be taken into account, for example, by authors and committees responsible for producing clinical practice guidelines relating to these conditions. One example of such knowledge translation are guidelines pertaining to the diagnosis of hypertension in pregnancy, which acknowledge the importance of posture in taking accurate blood pressure measurements.

Another common thread we observed was comparison of posture-physiology interactions in preeclampsia *versus* normotensive pregnancies. Pregnancies affected by preeclampsia, perhaps as a result of widespread endothelial dysfunction, volume contraction, and sympathetic overactivity, seem to be more sensitive to the effect of posture on physiology and have aberrant responses to orthostatic stress. This work is exciting, and its results could have both diagnostic and therapeutic applications in preeclampsia (Lewinsky and Riskin-Mashiah, 1998; Qureshi et al., 2019). We also encountered novel work relating to the possible contribution of posture to preeclampsia pathogenesis and disease activity via increased intra-abdominal pressures (Zhang, 2015), compression of retroperitoneal structures including the left renal vein at the aortic crossway (Tokunaga et al., 2000), and a condition dubbed, “renal compartment syndrome” (Reuter et al., 2016). While more research is needed regarding the effects of posture in preeclampsia, we point out the need for inclusion of other pregnancy-specific conditions in this area of research such as gestational diabetes.

The vast majority of research into posture-physiology interactions in pregnancy focuses on two systems: the musculoskeletal system and the circulatory system. This focus is understandable given the prominent role the musculoskeletal system plays in withstanding the effects of gravity and given the critical role the circulatory system plays in sustaining life. However, we encourage researchers to investigate posture-physiology interactions in other less-studied organ systems and have highlighted the gastrointestinal system as a gap in the literature. That said, our consideration of the corpus of evidence vis à vis posture-physiology interactions and the circulatory system (a relatively mature area of research) reveals underexplored research in other organ systems. For example, while limiting our scope to basic postures (i.e., left lateral, right lateral, supine, prone, sitting, and upright) would have provided ample material for discussion, some of the most interesting findings were gleaned from subtle variations in these basic postures, for example, various degrees of lateral tilt (Ellington et al., 1991; Bamber and Dresner, 2003; Kundra et al., 2012; Lee et al., 2012; Higuchi et al., 2015; Fujita et al., 2019). We advise others to pay close attention to these subtleties when both planning and executing their research protocols.

We conclude this discussion with additional research gaps and considerations. With regard to actual maternal postures under study, we found that inclusion of prone posture was rare (Oliveira et al., 2017; Dennis et al., 2018a). This is likely due to researchers’ altruistic intentions to avoid compressing the pregnant participant’s gravid uterus and fetus by her body weight and, thus, to avoid perceived harm; however, given the potential therapeutic benefit of the uterus resting on the anterior abdominal wall (as in a hammock) as it does in most quadrupeds, researchers should seek out methods to support the participant’s body while prone such that their weight is not compressing their uterus. Furthermore, another common theme we identified was that posture-physiology interactions are not static – they change across the trimesters as pregnancy advances. We commend the few groups that embarked on longitudinal studies of these interactions in the same participants from the first trimester through postpartum (Drinkwater and Chesnut, 1991; Schneider et al., 1993; Harirah et al., 2005; Vico et al., 2018; Alcahuz-Griñan et al., 2021). We found these studies to be least discrepant and most helpful in teasing apart the dynamicity of these interactions across the trimesters. As such, going forward, we discourage cross-sectional designs and encourage longitudinal work despite their increased complexity and expense. Finally, while the bulk of the literature we reviewed was complimentary and corroborating, we did uncover apparent discrepancies pertaining to some posture-physiology interactions. We feel that the bulk of these discrepancies likely stem from differences in study methodology, some of which was unclear in published reports, particularly the earlier literature. Leaders wishing to advance this research would benefit from collaborations to establish study protocols in order to harmonize the efforts of the field as a whole.

## Figures and Tables

**Table:** 

**AHI**	Apnea-hypopnea index
**ANP**	Atrial natriuretic peptide
**ANS**	Autonomic nervous system
**BMD**	Bone mineral density
**BMI**	Body mass index
**BNP**	Brain natriuretic peptide
**BP**	Blood pressure
**CO**	Cardiac output
**DBP**	Diastolic blood pressure
**DVT**	Deep vein thrombosis
**FPI**	Foot posture index
**FRC**	Functional residual capacity
**GERD**	Gastroesophageal reflux disease
**GH**	Gestational hypertension
**HDP**	Hypertensive disorders of pregnancy
**HR**	Heart rate
**HRV**	Heart rate variability
**HVP**	Hepatic venous pulsatility
**IAH**	Intra-abdominal hypertension
**IAP**	Intra-abdominal pressure
**ICC**	Intraclass correlation coefficient
**IOP**	Intraocular pressure
**IVC**	Inferior vena cava
**LBP**	Low back pain
**LE**	Lower extremity
**MAP**	Mean arterial pressure
**MG**	Myasthenia gravis
**MHM**	Mandibular hypermobility
**OPP**	Ocular perfusion pressure
**OSA**	Obstructive sleep apnea
**PAO**	Pregnancy-associated osteoporosis
**PaO2**	Arterial partial pressure of oxygen
**PDPH**	Postdural puncture headache
**PEFR**	Peak expiratory flow rate
**PGP**	Pelvic girdle pain
**POTS**	Postural orthostatic tachycardia syndrome
**RAAS**	Renin-angiotensin-aldosterone system
**RCT**	Randomized controlled trial
**RR**	Respiration rate
**SAP**	Systolic arterial pressure
**SBP**	Systolic blood pressure
**SG**	Striae gravidarum
**SHS**	Supine hypotensive syndrome
**SJH**	Systemic joint hypermobility
**SpO2**	Oxygen saturation
**SV**	Stroke volume
**SVR**	Systemic vascular resistance
**TOH**	Transient osteoporosis of the hip
