# Supplementary material for: Maternal posture-physiology interactions in human pregnancy: a narrative review
Source: Front Physiol. 2024 Jul 19;15:1370079. doi: 10.3389/fphys.2024.1370079 (PMC11294255; doi:10.3389/fphys.2024.1370079)
Supplement: Supplementary file 1 [file DataSheet2.docx]

**Supplement 2 – Musculoskeletal Maternal Posture-Physiology Interactions**

The musculoskeletal system performs several vital functions. One vital function of the musculoskeletal system is to withstand gravitational force, and at the same time, gravitational force is critical for the development and maintenance of the musculoskeletal system. Hence, pregnancy-induced changes in mass and moment arms portend posture-physiology interactions between pregnancy and the musculoskeletal system from head to toe.

## Head

In healthy pregnancies, the position of the head changes during standing. However, the direction and magnitude of the position change is not consistently reported in the literature. While Franklin and Conner-Kerr demonstrated a more posterior displacement of head position as pregnancy advances,[(1)](https://www.zotero.org/google-docs/?zzKCRL) Silveira *et al.* demonstrated anterior protrusion of the head as a result of the forward shift in the center of gravity.[(2)](https://www.zotero.org/google-docs/?9qRfAl) Head protrusion can affect the resting posture of the mandible, and increased mechanical loading along with hormonal changes of pregnancy can affect connective tissue laxity. Indeed, the authors observed mild mandibular hypermobility (MHM) in about 45% of pregnancies for jaw opening and lateralization to the left and right.[(2)](https://www.zotero.org/google-docs/?WXalhH) They also reported mild systemic joint hypermobility (SJH) in 50% of pregnancies in the second and third trimesters, but no association between SJH and MHM was consistently observed.[(2)](https://www.zotero.org/google-docs/?ed3vwS)

## Low Back and Pelvic Girdle

As pregnancy progresses, low back pain (LBP) and pelvic girdle pain (PGP) become increasingly common with a prevalence of around 40%.[(3–5)](https://www.zotero.org/google-docs/?q7SW4p) Despite its high prevalence, the pathophysiology is not well understood. Several factors have been associated with increased risk of LBP during pregnancy, including low socioeconomic status, LBP prior to the pregnancy, LBP in a previous pregnancy or between pregnancies.[(4,6)](https://www.zotero.org/google-docs/?RvGCg4) Studies have demonstrated the rate and severity of LBP and PGP increases with gestational age suggesting that asymmetrical forces exerted by the gravid uterus play a role in the development of LBP.[(3–5)](https://www.zotero.org/google-docs/?G6SGBd) Other risk factors include placental location – particularly, incidence of LBP increases when the placenta is in a posterior-fundal location,[(6)](https://www.zotero.org/google-docs/?5PcyQj) but a mechanistic link remains elusive. Regardless, the role of asymmetrical forces in LBP is further reinforced by studies which have not found an association between BMI and the prevalence of LBP.[(4,6)](https://www.zotero.org/google-docs/?QFiyKW)

In the third trimester of pregnancy, both sitting and standing become challenging and are associated with LBP.[(4,7)](https://www.zotero.org/google-docs/?yGSxkZ) Prolonged standing is also associated with increased PGP.[(3)](https://www.zotero.org/google-docs/?xByUsm) Sipko *et al.* found that due to increased pain, up to 40% of pregnant people are unable to sit for more than an hour, and many (36%) are unable to stand for more than half an hour.[(8)](https://www.zotero.org/google-docs/?8cgckC) Fung *et al.* also found posture impacted the severity of pain in pregnancy, with some patients finding relief by pacing (92%), assuming the decubitus (48.6%), supine (14.7%), or sitting (12.8%) posture, and placing a pillow under the low back (11.0%).[(5)](https://www.zotero.org/google-docs/?xmeCiu) On the other hand, supine (30.3%), standing (16.5%), and sitting (14.7%) posture were found to be aggravating factors for some patients. Ronchetti *et al.* found that there is a progression of difficulties with daily activities in PGP (least to most difficult): lying, sitting, walking, cycling, standing still.[(9)](https://www.zotero.org/google-docs/?aor9vU)

The study by Fung *et al.* also found that this pain resolves spontaneously in 62.4% of patients within the first 48 hours postpartum.[(5)](https://www.zotero.org/google-docs/?3AZx1l) In the immediate postpartum period, pain triggered by sitting is likely coccydynia, which is often accompanied by dislocation of the sacrococcygeal joint and is more prevalent in forceps- and vacuum-assisted births alongside spontaneous vaginal births described as “difficult”.[(10)](https://www.zotero.org/google-docs/?U1X2RV) New onset of long-term backache lasting more than six months postpartum tends to be postural, not severe, and more common in those who received epidural anesthesia in labor.[(11)](https://www.zotero.org/google-docs/?m3TUWg) Prenatal risk factors for development of postpartum LBP include more sitting time and less physical activity.[(12)](https://www.zotero.org/google-docs/?vhZVzE)

A meta-analysis of interventions for preventing and treating pelvic and back pain in pregnancy did not find any studies for pain prevention;[(13)](https://www.zotero.org/google-docs/?n75Xwt) however, of interest to a postural and gravitational lens, they found benefit of strengthening exercises, stretching exercises, sitting pelvic tilt exercises, and water gymnastics for reducing pain intensity and pain-related sick leave compared to usual prenatal care alone. They also reported in one study [(14)](https://www.zotero.org/google-docs/?mTjGGX) that found that a wedge-shaped pillow used to support the pregnant abdomen while lying on the side was more effective in relieving pain compared to a standard pillow.It is controversial whether postural adaptations of pregnancy contribute to pain.[(1)](https://www.zotero.org/google-docs/?JK5Z0J) It is also controversial what postural adaptations occur in pregnancy, which we will discuss below.

## Vertebral Column and Spine

Two groups investigating the lumbar spine curvature failed to confirm any changes in pregnancy.[(7,15)](https://www.zotero.org/google-docs/?pnY1Pd) However, other groups have demonstrated that spinal posture changes toward a tendency for decreased spinal curvature manifested as lumbar kyphosis (i.e., a decrease in lumbar lordosis) and sacral posterior inclination.[(1,16)](https://www.zotero.org/google-docs/?3oVktQ) In addition, Betsch and colleagues reported a significant increase in thoracic kyphosis and significant decrease in lateral deviation of the spine throughout the course of pregnancy.[(15)](https://www.zotero.org/google-docs/?rvrpTb)

Pregnancy has been associated with large changes in mass distribution, specifically in the lower trunk mass.[(17)](https://www.zotero.org/google-docs/?wXdzRZ) These changes affect a variety of biomechanical properties, including moment of inertia, posture, center of gravity, and gait. Jensen *et al.* found that during sit to stand movement, increases in the lower trunk mass, which primarily act through the gravitational moment, impact the hip intersegmental moment.[(17)](https://www.zotero.org/google-docs/?UTtCj9) Changes in forward flexion were both greater and occurred earlier in the pregnancy in the seated position compared to the standing position.

In studying standing and recumbent lumbar lordosis in pregnancies with preeclampsia, Kanayama *et al.* found significantly more hypolumbarlordosis compared to controls without preeclampsia.[(18)](https://www.zotero.org/google-docs/?tLRO6S) With the roll-over test mentioned in the **Renal** section (a positive result occurs when there is a 20 mmHg or higher diastolic arterial pressure when supine compared to the lateral recumbent posture), hypolumbarlordosis was significantly correlated to the increase in diastolic pressure, and hypolumbarlordosis was associated with increased peripheral vascular resistance.[(18)](https://www.zotero.org/google-docs/?kwMbaR) The underlying mechanism of hypersympathetic activation is thought to be compression of the mesenteric ganglia, the aortic plexus, and the lumbar sympathetic trunks between the gravid uterus and the more anteriorly protruded L1-L3 lumbar vertebrae in hypolumbarlordosis.

In the third trimester of pregnancy, for the L1-2, L2-3, L3-4, and L4-5 intervertebral disc levels, Xu and colleagues showed that the axial cross-sectional area and transverse maximum diameter of the dural sac is significantly greater in the lateral posture compared to supine.[(19)](https://www.zotero.org/google-docs/?whKTxK) The anteroposterior maximum diameter of the dural sac at the L4-5 level is greater in the lateral posture compared to supine; however, there are no significant differences in this dimension at the other lumbar levels. On MRI, the cross-section of the dural sac appears oval in the lateral posture (longest dimension oriented mediolateral); however, when supine, the cross-section becomes compressed in the mediolateral direction and appears near circular. Accompanying this change in cross-sectional shape, and possibly causative of the change, are bilateral shrunken-appearing epidural venous plexus in the lateral posture and bilateral engorged epidural venous plexus in the supine posture. This effect may have implications for nocturnal, centrally-mediated, radicular, low back pain when supine as numerous studies have shown compression of the dural sac by the engorged epidural venous plexus when supine in late pregnancy and have attributed this engorgement to downstream occlusion of the IVC by the gravid uterus in this position.[(20–26)](https://www.zotero.org/google-docs/?gXAmCr) While awake, this LBP can occur within 20 minutes of assuming the supine position as noted by Aluş *et al.* who found backache to be a complaint in over 60% of their third-trimester participants.[(27)](https://www.zotero.org/google-docs/?K0A64w)

In the postpartum period, the body center of mass changes in the lateral and anterior, but not vertical, directions. While abdominal mass and fluid retention regress quickly, breast tissue and other fat deposits persist, and it is thought that increased postpartum breast mass may contribute to persistent lumbar lordotic posture.[(28)](https://www.zotero.org/google-docs/?uqk64V) It is thought that anthropometry, standing posture, and body center of mass continue to change for up to 28 weeks postpartum.[(28)](https://www.zotero.org/google-docs/?19g4qx) The immediate reversal of pregnancy-related spine changes along with a change in activity (caring for the infant) have been postulated in a rare case to have triggered a spinal intradural hematoma from a previously undiagnosed neuroma two days following an uncomplicated vaginal delivery.[(29)](https://www.zotero.org/google-docs/?OWGQhP)

Overall, there are interactions between gravity, posture, and the vertebral column in pregnancy. Areas for future research include how changes in spine curvature and mechanics might impact blood pressure and pain modulation in pregnancy.

## Pelvis

In the third trimester, Sipko *et al.* found that up to 70% and 46% of pregnant individuals have static alterations in alignment of the pelvis (specifically, the posterior superior iliac spines are not symmetrical) when standing and sitting, respectively.[(8)](https://www.zotero.org/google-docs/?CAggXX) Furthermore, they found that 36% of pregnancies have disorders of the sacroiliac or hip joints, and this prevalence increases depending on which position (e.g., sitting, standing, recumbent) the joint is assessed in and which clinical test is used, which may have practical implications for pregnancy physiotherapists and other clinicians.[(8)](https://www.zotero.org/google-docs/?UhXhge) Contrariwise, Betsch *et al.* did not find significant changes in pelvic position during pregnancy.[(15)](https://www.zotero.org/google-docs/?xXjXy1) An MRI-based study by Eshed *et al.* suggests that the mechanical forces and hormones during pregnancy may induce acute inflammation, including bone marrow edema, joint fluid, enthesitis, and capsulitis, surrounding and within the sacroiliac joints of pregnant individuals experiencing LBP and PGP, which highlights the anatomic and physiologic impact mechanical forces during pregnancy may have on the low back and pelvic region.[(30)](https://www.zotero.org/google-docs/?MfcjEl) Eshed *et al.* hypothesize that pregnancy may induce chronic sacroiliitis in those genetically prone to spondyloarthritis. Further research is needed to confirm or refute this.

## Maternal Support Garments

Several studies have investigated maternal support garments to support the abdomen and low back to counter the physical changes during pregnancy that may contribute to the development of LBP and PGP. Several forms of maternal support garments have been created, including panties/briefs, belts or girdles, cradle, and torso supports. In pregnancies with LBP, using a lumbar and abdominal support while sitting and standing may reduce this pain after two weeks of use and does not appear to acutely affect maternal or fetal hemodynamics during late pregnancy.[(31)](https://www.zotero.org/google-docs/?tzwBZf) Quintero-Rodriguez and Troynikov systematically reviewed studies investigating maternal support garments and concluded there is strong evidence for these garments reducing pain in the low back, pelvic girdle, posterior pelvis, pubic symphysis, and sacroiliac joints.[(32)](https://www.zotero.org/google-docs/?DmRoUZ) Additionally, improvements were seen in functionality and mobility, as well as a reduction in risk of falling during pregnancy. The garments are thought to act by stabilizing the pelvic area or by increasing body proprioception; however, the exact mechanism of action of these garments has yet to be elucidated and should be a focus of future work. In passing, note that at least two professional societies recommend that, due to the postural changes in pregnancy, physiotherapist-guided core stability training should be implemented to prevent and treat back and pelvic pain during and following pregnancy.[(33,34)](https://www.zotero.org/google-docs/?nxs9gM) In the majority of patients with postpartum PGP, a pelvic belt improves the ability to perform the active straight leg raise test.[(35)](https://www.zotero.org/google-docs/?cdZawc)

## Bone Mineral Density

During pregnancy, bone mineral density (BMD) in the femoral neck, radial shaft, and lumbar spine decrease; however, BMD in the tibia increases.[(36)](https://www.zotero.org/google-docs/?KhHhae) These changes may be as a result of changing mechanical stress, hormones, postures, activity level, and calcium and vitamin D supply and demand during pregnancy and breastfeeding postpartum. Two distinct clinical entities in pregnancy related to bone metabolism are transient osteoporosis of the hip (TOH) and pregnancy-associated osteoporosis (PAO). While TOH, characterized by hip pain and fracture, is associated with immobility during pregnancy (up to three-fold more frequent immobilization compared with healthy controls), severe dental problems, and lack of exercise in childhood, the etiology and underlying pathophysiological mechanisms of TOH are still unclear, and it is likely a multifactorial disease.[(36)](https://www.zotero.org/google-docs/?ltSeI5) PAO is more severe than TOH and is characterized by low BMD and significant height loss from fragility fractures of thoracic or lumbar vertebrae (particularly T11 through L2) during pregnancy.[(37,38)](https://www.zotero.org/google-docs/?rnFhBH) The etiology and underlying pathophysiological mechanisms of PAO also remain enigmatic.[(36)](https://www.zotero.org/google-docs/?atvVNQ)

## Feet

In a study of the effect of pregnancy on the feet, Vico Pardo *et al.* reported that height and foot size remains unchanged with advancing pregnancy.[(39)](https://www.zotero.org/google-docs/?hxJypH) In contrast, Alcahuz-Griñan *et al.* reported an increase in foot length in the third trimester, flattening of the plantar arch, and significantly increased incidence of cavus in the third trimester.[(40)](https://www.zotero.org/google-docs/?mlsGb4) These changes, combined with hormonal changes that affect the ligaments of the foot, result in a gradual increase in left and right foot pronation (left greater than right), especially toward the end of pregnancy and returning to neutrality postpartum, as reflected by the foot posture index (FPI, a six-item clinical assessment tool that evaluates the multisegmental nature of foot posture in all three planes).[(39,40)](https://www.zotero.org/google-docs/?ByCNA0) However, while the FPI for the right foot approached statistical significance for an association with onset of low back pain, these changes were otherwise not associated with onset of pain in the lower extremities according to Vico Pardo *et al.*[(39)](https://www.zotero.org/google-docs/?edbCkG) In contrast, Vitor de Sousa Oliveira *et al.* have found significant and positive correlation between hindfoot pressure and functional disability, a significant and negative correlation between plantar pressure in the forefoot of the right foot and functional disability, and a significant and positive correlation between the contact area on the left foot and right foot and low back pain intensity.[(41)](https://www.zotero.org/google-docs/?G1rsp0) These findings are partially corroborated by Karadag-Saygi *et al.* who reported that in addition to increased anterior-posterior direction postural sway, there is an increase in forefoot pressure in the right foot with standing and walking in the third trimester (compared to overweight controls), both floor contact time and forefoot contact time are increased during walking, and the latter is correlated with foot pain.[(42)](https://www.zotero.org/google-docs/?GokFKP)

In a controlled trial that randomized first trimester participants to self-selected usual footwear versus their self-selected usual footwear with custom-molded arch-supportive orthoses, Segal *et al.* found no significant difference between groups at baseline or follow-up (8 weeks postpartum) in static arch height index while sitting or standing, arch drop, arch rigidity, or center of pressure excursion index.[(43)](https://www.zotero.org/google-docs/?ZHSmkZ) However, note that while the intraclass correlation coefficient (ICC) for sitting arch height index has been found reasonable for clinical use during pregnancy, Harrison and McCrory’s investigation has cast doubt on the clinical utility of the arch height index while standing given a lower than adequate ICC for this measure, which may partially explain Segal *et al.*’s null findings.[(44)](https://www.zotero.org/google-docs/?Vf0aY1) In patients who lost arch height during a previous pregnancy, Rabe and colleagues demonstrated that this results in internal tibial rotation, a shift in the tibiofemoral articulation, increased stress on the anterior cruciate ligament and menisci, but no change in tibiofemoral contact stress.[(45)](https://www.zotero.org/google-docs/?hXtRSn)

References

[1. Franklin ME, Conner-Kerr T. An analysis of posture and back pain in the first and third trimesters of pregnancy. J Orthop Sports Phys Ther. 1998 Sep;28(3):133–8.](https://www.zotero.org/google-docs/?wAY8ld)

[2. Silveira EB, Rocabado M, Russo AK, Cogo JC, Osorio RAL. Incidence of systemic joint hypermobility and temporomandibular joint hypermobility in pregnancy. Cranio J Craniomandib Pract. 2005 Apr;23(2):138–43.](https://www.zotero.org/google-docs/?wAY8ld)

[3. Ceprnja D, Chipchase L, Fahey P, Liamputtong P, Gupta A. Prevalence and Factors Associated with Pelvic Girdle Pain During Pregnancy in Australian Women: A Cross-Sectional Study. Spine. 2021 Jul 15;46(14):944–9.](https://www.zotero.org/google-docs/?wAY8ld)

[4. Mohseni-Bandpei MA, Fakhri M, Ahmad-Shirvani M, Bagheri-Nessami M, Khalilian AR, Shayesteh-Azar M, et al. Low back pain in 1,100 Iranian pregnant women: prevalence and risk factors. Spine J Off J North Am Spine Soc. 2009 Oct;9(10):795–801.](https://www.zotero.org/google-docs/?wAY8ld)

[5. Fung BK, Kwong CM, Ho ES. Low back pain of women during pregnancy in the mountainous district of central Taiwan. Zhonghua Yi Xue Za Zhi Chin Med J Free China Ed. 1993 Feb;51(2):103–6.](https://www.zotero.org/google-docs/?wAY8ld)

[6. Orvieto R, Achiron A, Ben-Rafael Z, Gelernter I, Achiron R. Low-back pain of pregnancy. Acta Obstet Gynecol Scand. 1994 Mar;73(3):209–14.](https://www.zotero.org/google-docs/?wAY8ld)

[7. Glinkowski WM, Tomasik P, Walesiak K, Głuszak M, Krawczak K, Michoński J, et al. Posture and low back pain during pregnancy - 3D study. Ginekol Pol. 2016;87(8):575–80.](https://www.zotero.org/google-docs/?wAY8ld)

[8. Sipko T, Grygier D, Barczyk K, Eliasz G. The occurrence of strain symptoms in the lumbosacral region and pelvis during pregnancy and after childbirth. J Manipulative Physiol Ther. 2010 Jun;33(5):370–7.](https://www.zotero.org/google-docs/?wAY8ld)

[9. Ronchetti I, Vleeming A, van Wingerden JP. Physical characteristics of women with severe pelvic girdle pain after pregnancy: a descriptive cohort study. Spine. 2008 Mar 1;33(5):E145-151.](https://www.zotero.org/google-docs/?wAY8ld)

[10. Maigne JY, Rusakiewicz F, Diouf M. Postpartum coccydynia: a case series study of 57 women. Eur J Phys Rehabil Med. 2012 Sep;48(3):387–92.](https://www.zotero.org/google-docs/?wAY8ld)

[11. Russell R, Groves P, Taub N, O’Dowd J, Reynolds F. Assessing long term backache after childbirth. BMJ. 1993 May 15;306(6888):1299–303.](https://www.zotero.org/google-docs/?wAY8ld)

[12. Ha VVA, Zhao Y, Pham MN, Binns CW, Nguyen CL, Nguyen PTH, et al. Physical Activity During Pregnancy and Postpartum Low Back Pain: A Prospective Cohort Study in Vietnam. Asia Pac J Public Health. 2019 Nov;31(8):701–9.](https://www.zotero.org/google-docs/?wAY8ld)

[13. Liddle SD, Pennick V. Interventions for preventing and treating low-back and pelvic pain during pregnancy. Cochrane Database Syst Rev. 2015 Sep 30;(9):CD001139.](https://www.zotero.org/google-docs/?wAY8ld)

[14. Thomas IL, Nicklin J, Pollock H, Faulkner K. Evaluation of a maternity cushion (Ozzlo pillow) for backache and insomnia in late pregnancy. Aust N Z J Obstet Gynaecol. 1989 May;29(2):133–8.](https://www.zotero.org/google-docs/?wAY8ld)

[15. Betsch M, Wehrle R, Dor L, Rapp W, Jungbluth P, Hakimi M, et al. Spinal posture and pelvic position during pregnancy: a prospective rasterstereographic pilot study. Eur Spine J Off Publ Eur Spine Soc Eur Spinal Deform Soc Eur Sect Cerv Spine Res Soc. 2015 Jun;24(6):1282–8.](https://www.zotero.org/google-docs/?wAY8ld)

[16. Okanishi N, Kito N, Akiyama M, Yamamoto M. Spinal curvature and characteristics of postural change in pregnant women. Acta Obstet Gynecol Scand. 2012 Jul;91(7):856–61.](https://www.zotero.org/google-docs/?wAY8ld)

[17. Jensen RK, Doucet S, Treitz T. Changes in segment mass and mass distribution during pregnancy. J Biomech. 1996 Feb;29(2):251–6.](https://www.zotero.org/google-docs/?wAY8ld)

[18. Kanayama N, el Maradny E, Kajiwara Y, Maehara K, Tokunaga N, Terao T. Hypolumbarlordosis: a predisposing factor for preeclampsia. Eur J Obstet Gynecol Reprod Biol. 1997 Dec;75(2):115–21.](https://www.zotero.org/google-docs/?wAY8ld)

[19. Xu F, Liu Y, Wei Y, Zhao Y, Yuan H, Guo X. Differences in lumbar dural sac dimension in supine and lateral positions in late pregnancy: a magnetic resonance imaging study. Int J Obstet Anesth. 2016 May;26:19–23.](https://www.zotero.org/google-docs/?wAY8ld)

[20. Paksoy Y, Gormus N. Epidural venous plexus enlargements presenting with radiculopathy and back pain in patients with inferior vena cava obstruction or occlusion. Spine. 2004 Nov 1;29(21):2419–24.](https://www.zotero.org/google-docs/?wAY8ld)

[21. Fast A, Hertz G. Nocturnal low back pain in pregnancy: polysomnographic correlates. Am J Reprod Immunol N Y N 1989. 1992 Dec;28(3–4):251–3.](https://www.zotero.org/google-docs/?wAY8ld)

[22. Fast A, Weiss L, Parikh S, Hertz G. Night backache in pregnancy. Hypothetical pathophysiological mechanisms. Am J Phys Med Rehabil. 1989 Oct;68(5):227–9.](https://www.zotero.org/google-docs/?wAY8ld)

[23. Hirabayashi Y, Shimizu R, Fukuda H, Saitoh K, Igarashi T. Effects of the pregnant uterus on the extradural venous plexus in the supine and lateral positions, as determined by magnetic resonance imaging. Br J Anaesth. 1997 Mar;78(3):317–9.](https://www.zotero.org/google-docs/?wAY8ld)

[24. Hirabayashi Y, Shimizu R, Fukuda H, Saitoh K, Igarashi T. Soft tissue anatomy within the vertebral canal in pregnant women. Br J Anaesth. 1996 Aug;77(2):153–6.](https://www.zotero.org/google-docs/?wAY8ld)

[25. Takiguchi T, Yamaguchi S, Tezuka M, Furukawa N, Kitajima T. Compression of the subarachnoid space by the engorged epidural venous plexus in pregnant women. Anesthesiology. 2006 Oct;105(4):848–51.](https://www.zotero.org/google-docs/?wAY8ld)

[26. Onuki E, Higuchi H, Takagi S, Nishijima K, Fujita N, Matsuura T, et al. Gestation-related reduction in lumbar cerebrospinal fluid volume and dural sac surface area. Anesth Analg. 2010 Jan 1;110(1):148–53.](https://www.zotero.org/google-docs/?wAY8ld)

[27. Aluş M, Okumuş H, Mete S, Güçlü S. The effects of different maternal positions on non-stress test: an experimental study. J Clin Nurs. 2007 Mar;16(3):562–8.](https://www.zotero.org/google-docs/?wAY8ld)

[28. Catena RD, Campbell N, Wolcott WC, Rothwell SA. Anthropometry, standing posture, and body center of mass changes up to 28 weeks postpartum in Caucasians in the United States. Gait Posture. 2019 May;70:196–202.](https://www.zotero.org/google-docs/?wAY8ld)

[29. Tanaka H, Kondo E, Kawato H, Kikukawa T, Ishihara A, Toyoda N. Spinal intradural hemorrhage due to a neurinoma in an early puerperal woman. Clin Neurol Neurosurg. 2002 Sep;104(4):303–5.](https://www.zotero.org/google-docs/?wAY8ld)

[30. Eshed I, Miloh-Raz H, Dulitzki M, Lidar Z, Aharoni D, Liberman B, et al. Peripartum changes of the sacroiliac joints on MRI: increasing mechanical load correlating with signs of edema and inflammation kindling spondyloarthropathy in the genetically prone. Clin Rheumatol. 2015 Aug;34(8):1419–26.](https://www.zotero.org/google-docs/?wAY8ld)

[31. Beaty CM, Bhaktaram VJ, Rayburn WF, Parker MJ, Christensen HD, Chandrasekaran K. Low backache during pregnancy. Acute hemodynamic effects of a lumbar support. J Reprod Med. 1999 Dec;44(12):1007–11.](https://www.zotero.org/google-docs/?wAY8ld)

[32. Quintero Rodriguez C, Troynikov O. The Effect of Maternity Support Garments on Alleviation of Pains and Discomforts during Pregnancy: A Systematic Review. J Pregnancy. 2019 Aug 1;2019:e2163790.](https://www.zotero.org/google-docs/?wAY8ld)

[33. Britnell SJ, Cole JV, Isherwood L, Sran MM, Britnell N, Burgi S, et al. Postural health in women: the role of physiotherapy. J Obstet Gynaecol Can JOGC J Obstet Gynecol Can JOGC. 2005 May;27(5):493–510.](https://www.zotero.org/google-docs/?wAY8ld)

[34. American College of Obstetricians and Gynecologists. Back Pain During Pregnancy [Internet]. [cited 2022 Feb 5]. Available from: https://www.acog.org/en/womens-health/faqs/back-pain-during-pregnancy](https://www.zotero.org/google-docs/?wAY8ld)

[35. Mens JM, Vleeming A, Snijders CJ, Stam HJ, Ginai AZ. The active straight leg raising test and mobility of the pelvic joints. Eur Spine J Off Publ Eur Spine Soc Eur Spinal Deform Soc Eur Sect Cerv Spine Res Soc. 1999;8(6):468–73.](https://www.zotero.org/google-docs/?wAY8ld)

[36. Drinkwater BL, Chesnut CH. Bone density changes during pregnancy and lactation in active women: a longitudinal study. Bone Miner. 1991 Aug;14(2):153–60.](https://www.zotero.org/google-docs/?wAY8ld)

[37. Kovacs CS, Ralston SH. Presentation and management of osteoporosis presenting in association with pregnancy or lactation. Osteoporos Int J Establ Result Coop Eur Found Osteoporos Natl Osteoporos Found USA. 2015 Sep;26(9):2223–41.](https://www.zotero.org/google-docs/?wAY8ld)

[38. Jun Jie Z, Ai G, Baojun W, Liang Z. Intertrochanteric fracture in pregnancy- and lactation-associated osteoporosis. J Int Med Res. 2020 Feb;48(2):300060519858013.](https://www.zotero.org/google-docs/?wAY8ld)

[39. Vico Pardo FJ, López Del Amo A, Pardo Rios M, Gijon-Nogueron G, Yuste CC. Changes in foot posture during pregnancy and their relation with musculoskeletal pain: A longitudinal cohort study. Women Birth J Aust Coll Midwives. 2018 Apr;31(2):e84–8.](https://www.zotero.org/google-docs/?wAY8ld)

[40. Alcahuz-Griñan M, Nieto-Gil P, Perez-Soriano P, Gijon-Nogueron G. Morphological and Postural Changes in the Foot during Pregnancy and Puerperium: A Longitudinal Study. Int J Environ Res Public Health. 2021 Mar 2;18(5):2423.](https://www.zotero.org/google-docs/?wAY8ld)

[41. Vitor de Sousa Oliveira G, Dibai Filho AV, Dibai DB, de Maria Araujo Mendonça Silva F, da Cunha Araújo Firmo W, Amorim de Sousa Garcia R, et al. Correlation between baropodometric variables, disability, and intensity of low back pain in pregnant women in the third trimester. J Bodyw Mov Ther. 2021 Jan;25:24–7.](https://www.zotero.org/google-docs/?wAY8ld)

[42. Karadag-Saygi E, Unlu-Ozkan F, Basgul A. Plantar pressure and foot pain in the last trimester of pregnancy. Foot Ankle Int. 2010 Feb;31(2):153–7.](https://www.zotero.org/google-docs/?wAY8ld)

[43. Segal NA, Neuman LN, Hochstedler MC, Hillstrom HL. Static and dynamic effects of customized insoles on attenuating arch collapse with pregnancy: A randomized controlled trial. Foot Edinb Scotl. 2018 Dec;37:16–22.](https://www.zotero.org/google-docs/?wAY8ld)

[44. Harrison KD, McCrory JL. Caliper Method Versus Digital Photogrammetry for Assessing Arch Height Index in Pregnant Women. J Am Podiatr Med Assoc. 2016 Nov;106(6):406–10.](https://www.zotero.org/google-docs/?wAY8ld)

[45. Rabe KG, Segal NA, Waheed S, Anderson DD. The Effect of Arch Drop on Tibial Rotation and Tibiofemoral Contact Stress in Postpartum Women. PM R. 2018 Nov;10(11):1137–44.](https://www.zotero.org/google-docs/?wAY8ld)
