# Supplementary material for: Maternal posture-physiology interactions in human pregnancy: a narrative review
Source: Front Physiol. 2024 Jul 19;15:1370079. doi: 10.3389/fphys.2024.1370079 (PMC11294255; doi:10.3389/fphys.2024.1370079)
Supplement: Supplementary file 2 [file DataSheet1.docx]

**Supplement 1 – Search Strategy Keywords**

The following keywords related to pregnancy were searched using the .mp field: “preconception” or “fertility” or “fertilization”or “pregnancy” or “pregnant” or “gestation” or “matern*” or “postpartum” or “post-partum” or “postnatal”.

The pregnancy terms were linked to body position keywords using the AND operator.

The following keywords related to body position were searched using the .mp field: “gravity” or “gravitation” or “posture” or “sitting” or “standing” or “prone” or “supine” or “recumben*”, or “position adj4 body” or “position adj4 sleep*” or “position adj4 dominant” or “position adj4 resting” or “position adj4 pregnan*” or “position adj4 matern*”, or “position adj4 trimester” or “position adj4 gestation*”.
